# Machine learning-based approach for reduction of energy consumption in hybrid energy storage electric vehicle

**DOI:** 10.1038/s41598-025-11330-1

**Published:** 2025-08-11

**Authors:** T. Paulraj, Yeddula Pedda Obulesu

**Affiliations:** School of Electrical Engineering, Vellore Institute of Technology, Vellore, Tamil Nadu India

**Keywords:** Supercapacitor, Battery, Energy management, Machine learning, LSTM, ONNX, BEV and HBEV, Electrical and electronic engineering, Batteries, Supercapacitors

## Abstract

This research introduces a novel machine learning-based strategy for generating supercapacitor (SC) reference current to optimize energy distribution in Battery Electric Vehicles (BEV) and Hybrid Battery Electric Vehicles (HBEV). A Long Short-Term Memory (LSTM) neural network is trained using real-world drive cycle data and exported in Open Neural Network Exchange (ONNX) format for real-time deployment within a Simulink-based control environment. This enables adaptive SC current prediction to dynamically offload high transient loads from the battery. The system is modeled using the Nissan Sakura EV and evaluated under EUDC and IM240 drive cycles. With SC support, the EUDC cycle exhibits a 21.3% reduction in battery peak current, 18.1% reduction in peak power demand, and 5.75% lower battery energy consumption. In the IM240 cycle, peak battery current is reduced by 33.5%, peak power by 31.6%, and energy consumption by 12.36%. These improvements validate the proposed LSTM-ONNX framework in reducing battery stress, improving thermal performance, and enhancing energy efficiency. Additionally, SC assistance leads to smoother traction motor torque, reduced current ripple, and optimized power delivery. A comprehensive model is developed and tested in Simscape to confirm the real-time applicability of this data-driven control strategy for electric vehicles.

## Introduction

### Aims and motivation

The global shift toward sustainable transportation has driven significant advancements in electric vehicle (EV) technologies. Reducing reliance on fossil fuels and minimizing environmental impact have become major priorities in the automotive industry. While battery-powered EVs offer a cleaner alternative to conventional vehicles, they face challenges related to battery degradation, energy efficiency, and power management. Frequent charge-discharge cycles, high power demands, and regenerative braking contribute to battery stress, ultimately affecting lifespan and overall performance.

To address these limitations, hybrid energy storage systems (HESS) have emerged as a promising solution by integrating supercapacitors (SCs) alongside batteries. Supercapacitors are well-suited for handling high power transients, making them effective for applications such as acceleration and regenerative braking^1–3^. This combination not only improves power distribution but also reduces the load on the battery, enhancing its lifespan and efficiency^3,4^. However, managing the energy flow between the battery and supercapacitor in real-time requires an intelligent control strategy to ensure optimal power allocation.

### Literature review

Several researchers have explored energy management strategies, control methodologies, and power electronics topologies to enhance efficiency, regenerative braking capabilities, and lifespan of battery-supercapacitor-based HESS. One study investigated the use of a hybrid EMCABN-ROA technique to efficiently manage energy between batteries and supercapacitors, achieving higher regenerative braking efficiency and reducing energy losses to just 4.5%^5^. Another study explored adaptive fuzzy neural networks for power distribution, demonstrating enhanced energy recovery and battery lifespan^6,7^.

Recent developments have also focused on DC/DC converter topologies for improved energy distribution^8^. A particular investigation concluded that an RST-based polynomial control strategy outperformed traditional PI controllers, making it easier to implement on microcontrollers and DSP platforms^9,10^. Furthermore, a PSO-based fractional-order controller was explored for hybrid systems, demonstrating improved power efficiency and reduced battery degradation^11,12^.

Additionally, power split strategies play a crucial role in energy management. A modified low-pass filter (MLPF)-based power split strategy has been proposed to allocate power between the battery and supercapacitor based on the SC’s state of charge (SoC), effectively utilizing the SC’s high-power-density property^13^. Moreover, a multiple input-multiple output (MIMO) model-based robust optimal controller has been developed to track time-varying reference currents, maintain constant DC bus voltage, and compensate for power deficiency and losses under disturbances. Simulation studies demonstrate excellent performance in terms of tracking accuracy, robustness to parameter variations, and power loss compensation under urban dynamometer driving schedules (UDDS). However, prior nonlinear controllers have faced challenges in terms of complexity and computational burden, limiting their real-time implementation^14^.

Another approach employs convex optimization techniques to enhance power allocation efficiency in battery- supercapacitor energy storage systems. A proposed optimization framework significantly reduces several battery degradation metrics compared to conventional low-pass filter approaches^15^. The alternating direction method of multipliers (ADMM) algorithm used for optimization achieves real-time computation, making it a promising candidate for online control. Despite these benefits, robustness in predicting errors and uncertainties in driver behavior require further investigation.

The impact of modeling approaches has also been a significant focus of research. A study introduced a polynomial capacitance-based SCAP model, which effectively captured the charge-discharge characteristics of supercapacitors. However, certain limitations persist in fully characterizing SC behavior before and after charge/discharge cycles^16^. Additionally, an adaptive PMP algorithm for HESS energy management was proposed, achieving computational efficiency 548 times higher than conventional dynamic programming (DP) while maintaining optimal performance^17–19^.

An intelligent energy management strategy (IEMS) based on linear parameter varying model predictive control (LPV-MPC) has been demonstrated to reduce battery degradation by minimizing root-mean-square battery current, discharge/charge peak current, ampere-hour throughput, capacity loss, and energy loss^20^. This approach incorporates anticipated acceleration-based control for further enhancement. However, real-world testing on physical battery packs remains a challenge, and current evaluations are limited to a handful of degradation metrics.

Hybrid energy storage system configurations have shown promising results in reducing battery voltage drops, peak currents, energy capacity loss, and fading cost across various driving cycles. Studies indicate that HESS reduces battery voltage drops by 22.73–36.36%, peak battery currents by 28.81–40% compared to sole battery storage systems^21^.

Furthermore, an adaptive disturbance suppression composite disturbance observer-based (ADSCDOB) control scheme has been introduced for HESS to counteract uncertainties under complex driving conditions^22^. This approach avoids differential explosion problems associated with prior adaptive backstepping control techniques while ensuring stability. Simulation and prototype experiments confirm its effectiveness in enhancing response time, reducing error, and improving stability under hybrid driving conditions. Future research should consider uncertainty management, converter power loss analysis, regenerative braking integration, and extending this control strategy to other energy storage applications such as microgrids and rail transit.

Regarding experimental validation, researchers have compared finite element analysis (FEA) models with practical test bench measurements, confirming the accuracy of simulation-based performance predictions. Hardware-in-the-loop (HIL) testing has also been incorporated into recent studies, further refining real-time energy management implementations. However, some studies faced cost constraints, leading to scaled-down experimental setups^23–25^.

Traditional rule-based energy management strategies often rely on fixed thresholds, which may not adapt effectively to dynamic driving conditions^26–28^. With recent advancements in artificial intelligence (AI) and machine learning (ML), data-driven approaches have been explored for real-time control in EV power systems. Machine learning-based control enables adaptive decision-making, optimizing energy flow between storage elements while considering dynamic driving conditions^29,30^.

Optimization techniques like the Red-Tailed Hawk Algorithm-optimized Artificial Neural Network (RTHA-ANN)^31^, Squirrel Search with Improved Food Storage (SS-IFS)^32^, and Hybrid Bat Algorithm-enhanced RNNs (HBA-RERNN)^33^ have shown improved performance in power control, voltage stability, and SC sizing for EV applications. Deep learning approaches using DNNs, Recurrent Neural Networks (RERNN), and Zebra Optimization Algorithms (ZOA)^34^ have also enhanced prediction accuracy under dynamic driving loads but still face challenges in computational complexity and scalability.

Driving pattern recognition (DPR) is another active domain, with studies employing ANFIS-based strategies^35^, adaptive ECMS, and ensemble learning velocity predictors^36^ to adjust energy flow according to driver behavior. These methods reduce battery stress and improve energy efficiency but often involve high offline computational costs, making real-time deployment difficult. Meanwhile, VMD-ELM models^37^ and stochastic design frameworks further demonstrated efficiency in handling uncertain conditions like variable driver profiles and vehicle mass.

Regenerative braking optimization has also been widely studied. A simplified RBS using battery/SC hybrids achieved ~ 20% higher energy recovery^38^, while control strategies based on Passivity-Based Control (IDA-PBC) with ANN^39^and driving-style-aware ECMS^40^ demonstrated reduced fuel and power demands. Other works, such as^41^, addressed the influence of SC sizing and intensity factors in city buses, revealing that standardized EMS schemes can be generalized across similar drive cycles.

Machine learning-driven energy control has gained traction in recent works, with ANN-based hybrid controllers^31^, Deep Forecasting Networks, and NARX neural models^42^ deployed to manage energy across varied driving scenarios. However, many of these methods, including AI-based HPESS control, exhibit limited adaptability when deployed in unpredictable environments or embedded systems.

Table-based comparative studies, sensitivity analyses, and multi-objective sizing using evolutionary algorithms^43^ reveal that although system-level optimizations can reduce battery degradation, most frameworks either lack real-time execution capability or remain untested in closed-loop hardware-in-the-loop environments. Furthermore, limitations such as power converter losses, electromagnetic interference, and the challenge of accurate SC modeling during transients persist.

Although recent studies—such as the integration of decentralized controllers^44^ and adaptive dynamic surface control^22^ have improved real-time behavior and tracking accuracy, research gaps remain. Notably, most models lack compatibility with platforms like MATLAB/Simulink, especially for real-time inference using pre-trained neural networks. Very few works demonstrate practical deployment of Python-trained machine learning models in Simulink via ONNX, where bidirectional data integration and real-time prediction are critical. Moreover, thermal analysis of battery relief under SC support, though critical, is rarely explored alongside electrical performance. The Comparative summary of recent ML-based energy management strategies for electric vehicle hybrid energy storage systems is shown in Table 1.

**Table 1:** Summary of recent ML-based energy management strategies for electric vehicle hybrid energy storage systems.

Ref	Method/Approach	Main contributions	Limitations
^35^	ANFIS + DPR	Reduces RMS battery current; evaluates SC sizing & SoC variation.	High computational effort; data sensitivity.
^6^	AFNN + Clustering	Power sharing, battery stress reduction, peak battery current reduction (37.4%).	Difficulty in Rule Base Optimization; increases computational load.
^45^	SC buffering + ML logic	18% cost reduction in battery aging; improved SC coordination.	Centralized-only; battery stress due to cycling.
^46^	HWT + DPR	25.2% peak current drop; 6.16% battery life gain.	Offline processing; high online cost.
^39^	ANN + PBC	Power sharing, peak current drop (33.2%); RMS current drop (9.12%).	Limited in real world scenario, large dataset required for training.
^40^	Adaptive ECMS + style recognition	3.69% energy economy gain; validated on real PHEV.	Limited transferability to other PHEV.
^43^	GWO-SVM	Reduces energy loss (17.9%), peak current drop (38.2%) and RMS current (8.8%).	High data sensitivity; SC lifecycle modeling incomplete.
^47^	DNN + Meta-heuristic control	Prolongs battery life, stress reduction; hybrid control via optimization.	High algorithmic complexity: some methods lack pattern adaptation.
^22^	Adaptive DSC + DOB	Fast, robust control under hybrid load; battery stress reduction.	More computational time and high regenerative loss.
^48^	Hybrid optimization + ML	Minimizes battery degradation; cost-optimized sizing.	Requires case-by-case tuning; lacks modular scalability.
^38^	RBS + NN	20% regenerative efficiency boost; extended EV range.	Cost inefficiency: SC utilization limited; voltage constraints.
^49^	V2C + EMS	13.89% less battery degradation; V2C adds 6.81% more savings.	DP and MPC are too computationally heavy; rule-based lacks adaptability.
^42^	NARX-NN + DIFESS	Enhanced usable capacity (up to 26.2%); effective capacity control.	Assumptions in system modeling; challenges in adaptive control under real conditions.
^34^	Zebra Optimization + RERNN	Superior DC voltage regulation; reduced mean error.	High complexity; scalability and real-time control issues.
^50^	ML + Dynamic surface control	Maintains accurate tracking, good battery health.	Does not address real converter losses or temperature effects.
^51^	Adaptive ECMS + Driving style	Fuel economy up to 9.54%; real-time HIL tested.	Simulation-only unpredictability lacks wide validation.
^52^	Hybrid RNN + Optimization	Efficiency ~ 94.8%; reduced degradation and cost.	Fuzzy logic & GA complexity; topology analysis incomplete.
^53^	KHO-RDF + FLC	Improves SC voltage, reduces power mismatch.	Heuristic tuning problems, lack real-time feasibility.
^36^	Ensemble ML + SOC reference	28.88% fuel savings vs. CD-CS; near-global optimal performance.	DP still costly; optimization burden remains
^54^	DPR + PMP + DFSS	34.36% fuel economy improvement; robust against vehicle mass noise.	Complex control for online mass evaluation; recognition failures possible.
^55^	AECMS + VMD-ELM	6% fuel savings; accurate driving pattern classification (96.67%).	Limited analysis of EMS link for complex cycles.
^39,56^	ANN + Passivity-Based Control	Real-time operation; improves SC & battery coordination.	Needs experimental thermal validation; single SC stack focus.
^57^	DRL-based multi-Agent control	Aligns SC size to cycle intensity; enhanced regenerative braking.	DP-based EMS impractical in real-time.
^31^	RTHA-optimized ANN	Enhanced predictive control under varying drive cycle; validated on prototype.	Complex control; static loads; high ANN data demand.
^58^	WT + FLC	Peak discharge current reduction (58.2%), RMS current reduction 11.2%	High computational time, real time control issues.
CP	ONNX-integrated LSTM	Reduces battery peak current, voltage ripple, power demand, thermal stress, energy consumption and traction motor efficiency.	Trained model performance depends on the quality and range of drive cycle data.

CP-Current Paper.

### Research gaps and contributions

Although numerous studies have explored HESS for electric vehicles using machine learning, meta-heuristic, and rule-based energy management strategies, several significant challenges persist^59^:

- High computational cost and limited real-time feasibility: Many optimization-based methods (ANN, DNN, ANFIS, DP, MPC, GA, Fuzzy, and SVM + PSO) suffer from high computational demands, limiting practical deployment in embedded automotive platforms.
- Lack of real-time adaptability to dynamic driving conditions: Most methods rely on offline processing or static rule-based thresholds, which fail to adapt to varying speed, load, and terrain patterns experienced in real-world conditions.
- Limited generalization and dependency on extensive training data: Several models, especially ANN and fuzzy-logic-based EMS, struggle to generalize across diverse drive cycles, often requiring re-training or manual tuning for each scenario.
- Neglect of practical implementation pathways: While some strategies achieve promising simulation results, few integrate models into real-time platforms like MATLAB Simulink with ONNX deployment, limiting reproducibility and industrial relevance.
- Thermal and voltage performance underexplored: Many studies emphasize current or SoC tracking accuracy, while the effect on battery thermal behavior, voltage ripple, and power demand fluctuation remains underreported.
- Inadequate focus on practical system integration: Issues like bidirectional converter efficiency losses, real-time sensor interfacing, and SC utilization during regenerative braking are often idealized or simplified.

To bridge these gaps, this paper proposes a practical, ML-integrated energy management system tailored for hybrid electric vehicles with the following innovations:

- Hybrid energy storage control using ONNX-deployed deep learning: A novel Long Short-Term Memory (LSTM)-based SC reference current prediction model is developed and exported via ONNX format, enabling real-time use in MATLAB Simulink for seamless integration into vehicle simulation frameworks.
- Drive cycle-informed, data-driven control: The model learns SC current demand patterns from real-world driving data (EUDC and IM240) using acceleration and velocity inputs, allowing adaptive behavior across multiple scenarios without manual rule tuning.
- Reduced battery stress and improved energy efficiency: By offloading transient power demands to the SC, the system significantly mitigates battery peak current, stabilizes voltage, and improves energy usage efficiency compared to conventional BEV configurations.
- Quantitative performance validation with multi-metric error analysis: RMSE, MAE, MAPE, and R² values confirm that the LSTM-based SC current generation closely matches analytical results under dynamic operating conditions, supporting model reliability.
- Simulink-based implementation and comparison with analytical approach: The framework bridges theoretical modeling and practical application by comparing equation-based and LSTM-predicted SC currents, validating the ML approach with near-identical trajectories and minimal deviation.
- Thermal and voltage impact studies integrated into drive cycle simulation: Battery thermal behavior and voltage performance are included in the comparative evaluation, highlighting real-world benefits of SC-assisted operation under transient loading.
- Relevance to industry and embedded deployment: The use of ONNX enables portability across embedded controllers, aligning the proposed system with deployment-ready architectures suitable for automotive-grade implementations.

**Fig. 1:**
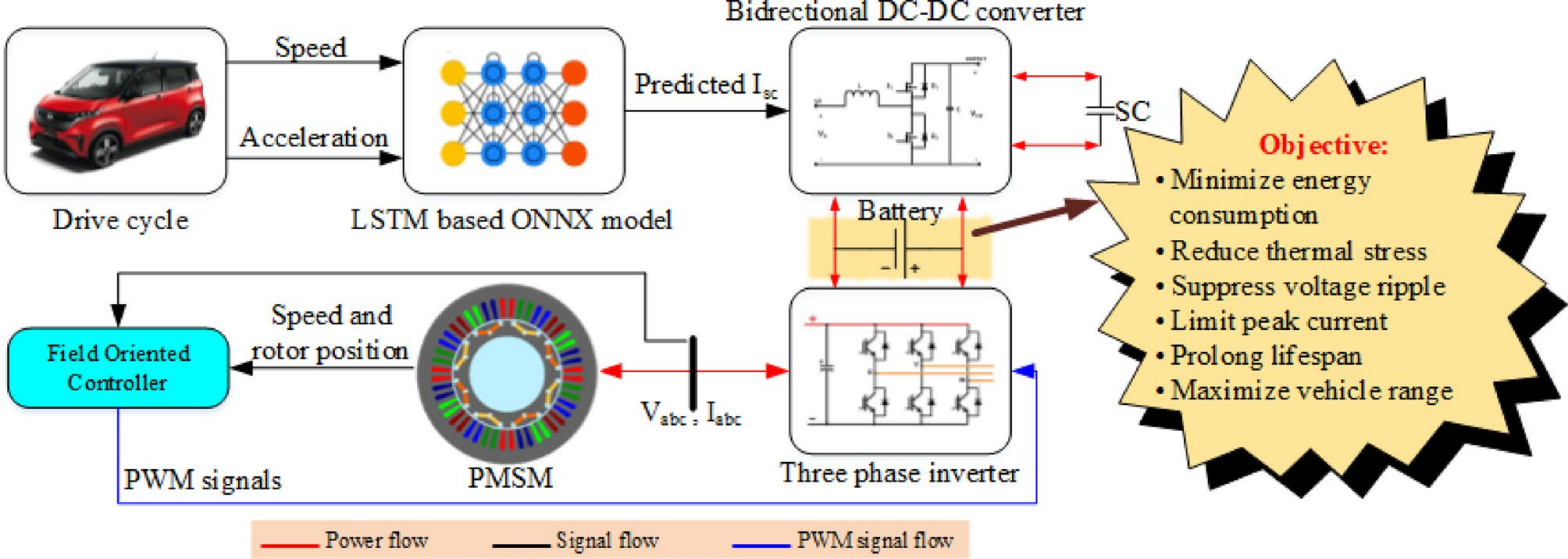
Graphical abstract of the proposed ONNX-deployed LSTM-based SC current prediction framework for EVs.

To visually summarize the key innovations and workflow of the proposed system, the graphical abstract shown in Fig. 1 illustrates the integration of the ONNX-deployed LSTM model for real-time SC current prediction, its interaction with the EV powertrain, and the overall objective of improving battery performance and energy efficiency.

### Paper organization

Section 2 outlines the system architecture of the HESS. Section 3 explains the traction motor selection based on vehicle dynamics. Section 4 presents the analytical sizing of the battery and SC. Section 5 describes SC reference current generation using both an analytical approach and an LSTM-based ONNX model. Section 6 discusses the implementation of the proposed system and lastly Sect. 7 describes the results and key findings.

## System overview and energy management strategy

**Fig. 2:**
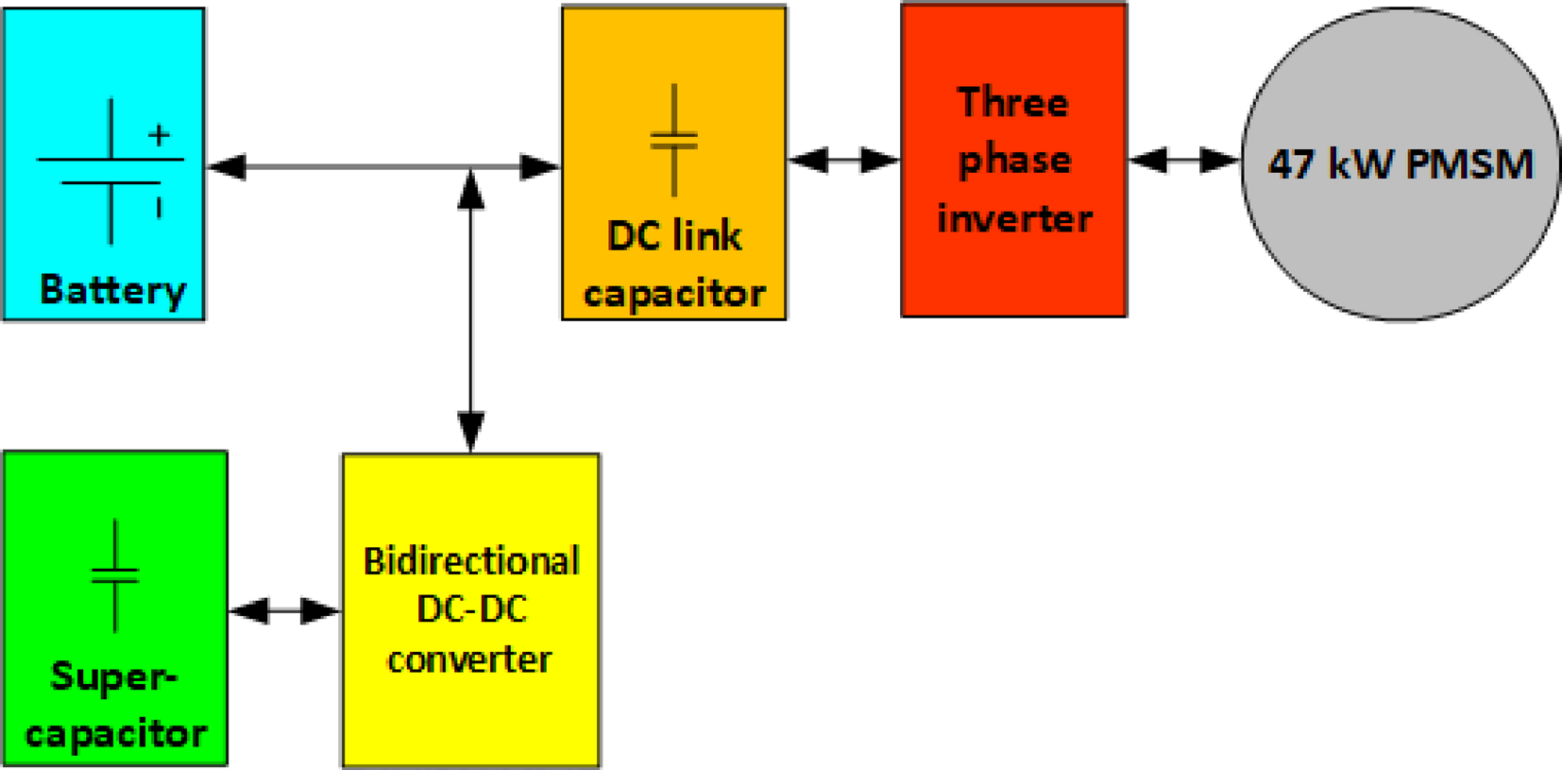
Block diagram of battery and supercapacitor energy management in PMSM based powertrain.

To achieve an efficient and reliable powertrain, a combination of a battery, supercapacitor, bidirectional DC-DC converter, DC-link capacitor, and three-phase inverter is used to drive the traction motor as shown in Fig. 2. The battery serves as the primary energy source, supplying continuous power for vehicle operation, while the supercapacitor supports transient power demands such as acceleration and regenerative braking. The bidirectional DC-DC converter regulates power exchange between the battery and the supercapacitor, ensuring optimal energy distribution^32,60,61^. The DC-link capacitor stabilizes voltage fluctuations before the three-phase inverter converts DC power into AC to drive the PMSM efficiently.

Given this powertrain architecture, the selection of the traction motor power rating is a crucial step in determining system performance, energy efficiency, and the sizing of energy storage components. The battery must be sized appropriately to provide the required steady-state power, ensuring sufficient capacity to support sustained driving conditions. Meanwhile, the supercapacitor is sized based on its ability to handle rapid power fluctuations, reducing stress on the battery and improving its lifespan. The following sections detail the process of selecting the traction motor power rating based on vehicle dynamics and establishing the appropriate sizing criteria for both the battery and the supercapacitor.

## Selection of traction motor rated power based on vehicle dynamics

**Fig. 3:**
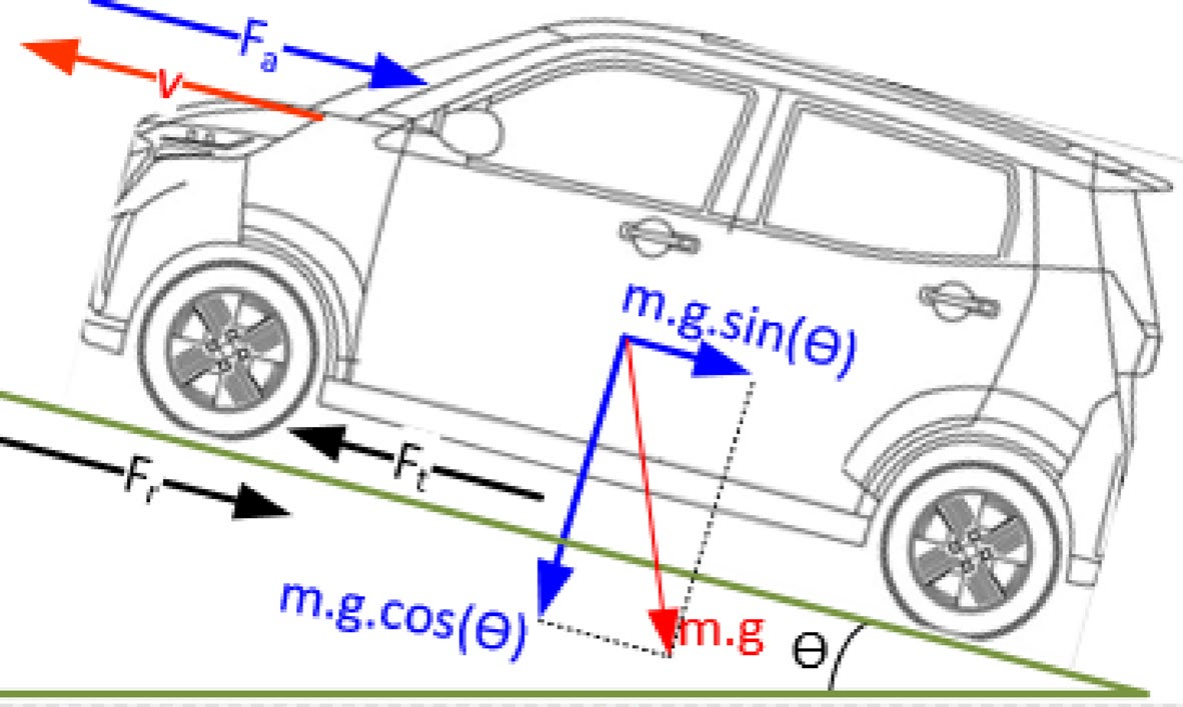
Vehicle longitudinal dynamics and force distribution.

The rated power of a traction motor in an EV is determined by several resistive forces acting on the vehicle, including aerodynamic drag, rolling resistance, gradient force, and inertial force during acceleration as shown in Fig. 3.

These forces influence the total traction power requirement, which dictates the motor’s rated power. The total traction force required to propel the vehicle can be expressed as^62,63^:

1 $$\:{\text{F}}_{\text{t}}=\:{\text{F}}_{\text{a}}+{\text{F}}_{\text{r}}+{\text{F}}_{\text{g}}+{\text{F}}_{\text{i}}$$.

Here, $\:{\text{F}}_{\text{a}}$ is the aerodynamic drag force, $\:{\text{F}}_{\text{r}}$ is the rolling resistance force, $\:{\text{F}}_{\text{g}}$ is the gradient force, $\:{\text{F}}_{\text{i}}$ is the force required for vehicle acceleration.

2 $$\:{\text{F}}_{\text{a}}=\:\frac{{\uprho\:}}{2}{.\text{C}}_{\text{d}}.{\text{A}}_{\text{f}}.{\text{v}}^{2}\:$$

3 $$\:{\text{F}}_{\text{r}}=\:{\text{C}}_{\text{r}\text{r}}.m.\text{g}\:.\text{c}\text{o}\text{s}\left({\uptheta\:}\right)$$

4 $$\:{\text{F}}_{g}=\:m.\text{g}\:.\text{s}\text{i}\text{n}\left({\uptheta\:}\right)$$

5 $$\:{\text{F}}_{i}=\:m.\text{a}$$.

Here, $\:{\uprho\:}$ is the air density (kg/$\:{\text{m}}^{3}$), $\:{\text{C}}_{\text{d}}$ is the aerodynamic drag coefficient, $\:{\text{A}}_{\text{f}}$ is the frontal area of the vehicle ($\:{\text{m}}^{2}$), $\:\text{v}$ is the vehicle speed (m/s), $\:{\text{C}}_{\text{r}\text{r}}$ is the rolling resistance coefficient, $\:m$ is the vehicle mass (kg), $\:\text{g}$ is the gravitational acceleration (9.81 m/s²), $\:{\uptheta\:}$ is the road inclination angle (radians), $\:\text{a}$ is the vehicle acceleration (m/$\:{\text{s}}^{2}$). The total traction power ($\:{P}_{t}$) required by the motor is given by^44,64^:

).v (6)

6 $$\:{P}_{t}={F}_{t}.v\:=\:(\frac{{\uprho\:}}{2}{.\text{C}}_{\text{d}}.{\text{A}}_{\text{f}}.{\text{v}}^{2}+{\text{C}}_{\text{r}\text{r}}.m.\text{g}\:.\text{c}\text{o}\text{s}({\uptheta\:})+m.\text{g}\:.\text{s}\text{i}\text{n}({\uptheta\:})+m.\text{a}$$

## Energy storage system design for EV powertrain

Electric vehicles require an efficient energy storage system to meet varying power demands during different driving conditions. The battery serves as the primary energy source, supplying steady power for vehicle propulsion, while the supercapacitor is used to handle transient power demands during acceleration and regenerative braking. This section presents the selection criteria and sizing methodology for both battery and supercapacitor units based on vehicle dynamics and powertrain requirements.

### Battery sizing for EV powertrain

The battery selection process is primarily governed by the total energy requirement of the EV. Since the battery must supply power to the traction motor and auxiliary systems, its capacity must be sufficient to sustain operation over an entire drive cycle without excessive discharge. The total power required by the battery is calculated as:

7 $$\:{P}_{b}=\:\frac{{P}_{t}{+P}_{aux}}{{\eta\:}_{d}}$$.

Here $\:{\text{P}}_{\text{b}}$ is the battery power (W), $\:{\text{P}}_{\text{a}\text{u}\text{x}}$ is the power required for auxiliaries like lights, fan, controllers, AC and $\:{\eta\:}_{\text{d}}$ is the drivetrain efficiency, accounting for losses in the inverter, motor, and transmission.

8 $$\:{E}_{b}=\:\int\:{P}_{b}.t\:$$

9 $$\:{I}_{b}=\:\frac{{P}_{b}}{{V}_{b}}\:$$.

$\:{\text{E}}_{\text{b}}$ represents the total energy drawn from the battery in watt-hours (Wh). $\:{\text{V}}_{\text{b}}$ is the nominal voltage of the battery pack, $\:{\text{I}}_{\text{b}}$ is the battery current in A. To ensure sufficient storage for long-term operation, the required battery capacity is determined as^45,65^:

10 $$\:{C}_{b}=\:\frac{{E}_{b}}{{V}_{b}.DOD}\:$$.

Where $\:{\text{C}}_{\text{b}}$ represents the total charge, the battery can store (Ah) and DOD (Depth of Discharge) represents the fraction of total capacity utilized during operation. A typical DOD range of 70–90% is recommended to extend battery life.

### Supercapacitor sizing for transient power support

While the battery provides steady power, it is not ideal for handling rapid fluctuations in power demand. This limitation is addressed by integrating a supercapacitor, which delivers high bursts of power during acceleration and absorbs regenerative energy during braking. The energy stored in a supercapacitor is given by^66,67^:

11 $$\:{\text{E}}_{\text{s}\text{c}}=\:\frac{1}{2}{\text{C}}_{\text{s}\text{c}}\left({\text{V}}_{\text{s}\text{c}\_\text{m}\text{a}\text{x}}^{2}-{\text{V}}_{\text{s}\text{c}\_\text{m}\text{i}\text{n}}^{2}\right)$$.

Here $\:{C}_{sc}$ is the capacitance value of SC, $\:{V}_{\text{s}\text{c}\_\text{m}\text{a}\text{x}}$ is the maximum operating voltage of SC, $\:{V}_{\text{s}\text{c}\_\text{m}\text{i}\text{n}}$ is the minimum voltage after discharge. The supercapacitor supplies power based on its voltage and current:

12 $$\:{\text{P}}_{\text{s}\text{c}}=\:{\text{V}}_{\text{s}\text{c}}.{\text{I}}_{\text{s}\text{c}}$$.

Since the voltage across a supercapacitor varies dynamically, the instantaneous current is:

13 $$\:{\text{I}}_{\text{s}\text{c}}\:=\:-{C}_{sc}\frac{d{\text{V}}_{\text{s}\text{c}}}{d\text{t}}$$.

To size the supercapacitor appropriately, the required capacitance is computed using^68^:

14 $$\:{C}_{sc}=\:\frac{2.{E}_{sc}}{{\text{V}}_{\text{s}\text{c}\_\text{m}\text{a}\text{x}}^{2}-{\text{V}}_{\text{s}\text{c}\_\text{m}\text{i}\text{n}}^{2}}$$.

where $\:{\text{E}}_{\text{s}\text{c}}$​ is determined based on peak power requirements and energy recovery efficiency during braking.

## Supercapacitor reference current generation

Supercapacitor reference current generation plays a vital role in managing energy flow in HESS. Accurate prediction of this current ensures optimal coordination between the battery and the supercapacitor during high power transients such as acceleration and regenerative braking. Two methodologies are explored in this work: an analytical approach based on predefined current equations derived from drive dynamics and system constraints, and a data-driven method utilizing a LSTM neural network trained on historical drive cycle data. The analytical method provides interpretability and control transparency, while the LSTM-based model offers enhanced adaptability under dynamic conditions. The following subsections detail both techniques, highlighting their implementation and accuracy.

### Analytical approach for reference current generation

From the principle of energy conservation, the energy stored in the supercapacitor (SC) is related to the kinetic energy of the vehicle, which can be expressed as^38,42^:

15 $$\:\frac{1}{2}{C}_{sc}\left({V}_{sc\_max}^{2}-{V}_{sc\_ins}^{2}\right)=\frac{1}{2}m{.v}^{2}$$.

Here $\:{V}_{sc\_ins}$ is the instantaneous SC voltage after charge and discharge (V), m is the mass of the vehicle in kg and v is the velocity of the vehicle in m/s. After simplification, the instantaneous SC voltage can be expressed as:

16 $$\:{V}_{sc\_ins}=\sqrt{{V}_{sc\_max}^{2}-\frac{m{.v}^{2}}{{C}_{sc}}\:}$$.

During acceleration and deceleration, the SC injects and absorbs current from the traction motor. The rate of change of SC voltage determines the SC current, which is given by:

17 $$\:{I}_{sc}=\:-{C}_{sc}\frac{d{V}_{sc\_ins}}{dt}\:=\:-{C}_{sc}\frac{d\sqrt{{V}_{sc\_max}^{2}-\frac{m{.v}^{2}}{{C}_{sc}}\:}}{dt}$$.

After further simplification, the SC current can be expressed as:

18 $$\:{I}_{sc}=\:\frac{m.v}{{V}_{sc\_max}}.\frac{dv}{dt}.\sqrt{1-\frac{m{.v}^{2}}{{V}_{sc\_max}^{2}.{C}_{sc}}}$$.

This equation shows that the SC current is directly proportional to the acceleration and speed of the vehicle. If both speed and acceleration are zero, the SC current $\:{I}_{sc}$​ is also zero. Since the SC is primarily utilized during acceleration and deceleration, the computed $\:{I}_{sc}$​ can be considered as the SC reference current for the energy management system.

### Data-Driven (LSTM-Based) reference current prediction

To enhance the accuracy and adaptability of SC reference current generation, a deep learning approach based on a LSTM neural network has been implemented. The model is trained using vehicle acceleration, velocity, and corresponding SC current reference data, all extracted and preprocessed from a.mat file. The preprocessing stage includes normalization and noise filtering to ensure high-quality input features for effective learning.

The LSTM network architecture is developed in Python using the PyTorch framework. Owing to its ability to capture long-term dependencies and temporal dynamics in time-series data, the LSTM model is well-suited for predicting the SC current profile under varying drive cycle conditions. Once trained and validated, the LSTM model is exported to the Open Neural Network Exchange (ONNX) format for platform-independent deployment.

**Fig. 4:**
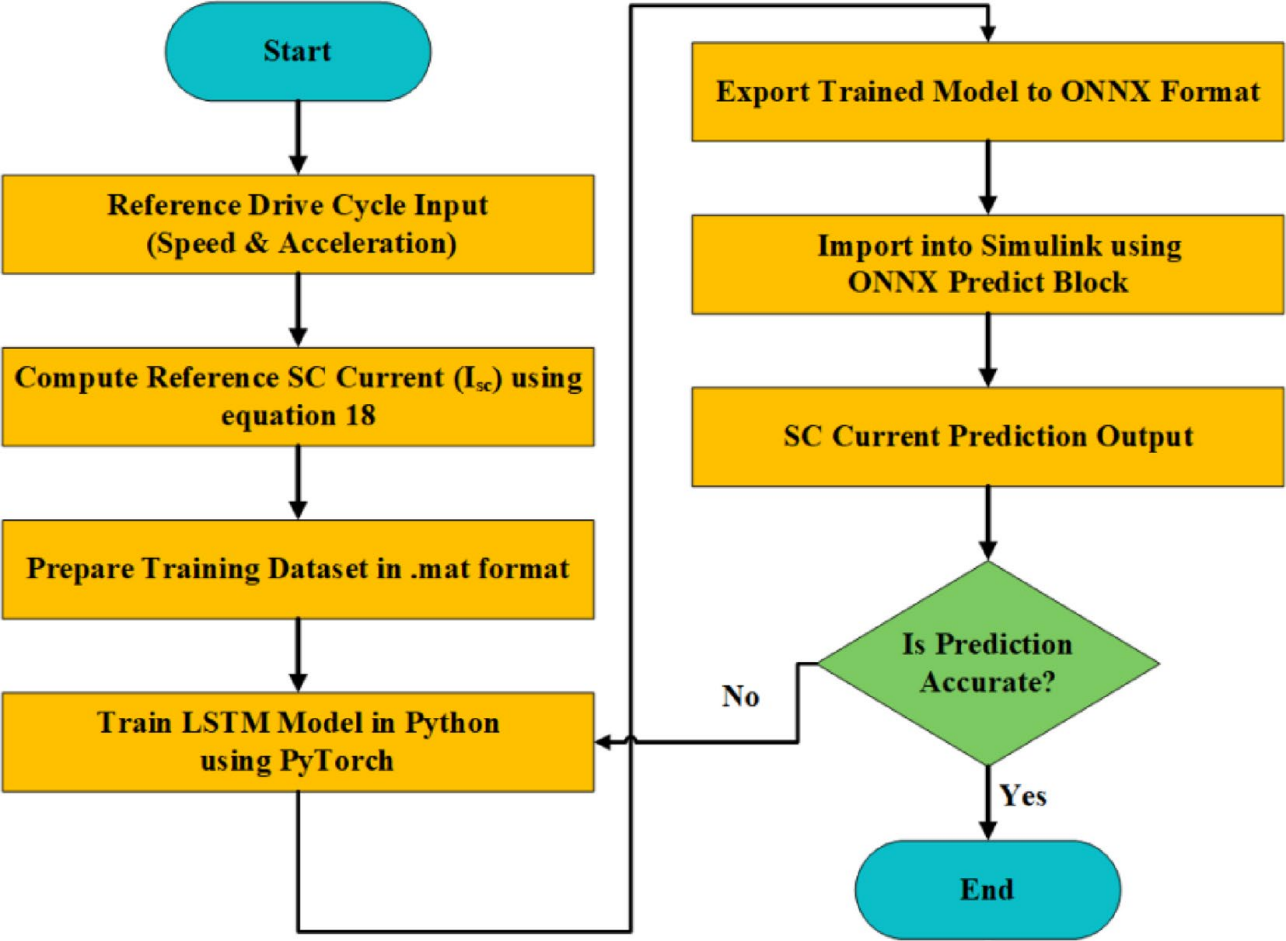
Flowchart of SC current reference generation using an ONNX-integrated machine learning model.

This ONNX model is then integrated into MATLAB Simulink using the ONNX Predict block, enabling real-time SC current prediction within the HESS control loop. As illustrated in Fig. 4, the ONNX-enabled LSTM model continuously estimates $\:{I}_{sc}$ based on instantaneous vehicle acceleration and speed, thereby offering a data-driven alternative to conventional equation-based reference generation techniques.

To ensure robust performance, several key hyperparameters of the LSTM model—such as learning rate, number of hidden units, batch size, activation function, and number of epochs—were empirically tuned through iterative experimentation. Table 2 summarizes the final hyperparameter values selected to achieve optimal convergence and generalization across both EUDC and IM240 drive cycle conditions.

**Table 2:** Selected hyperparameters for the SC current prediction model.

Hyperparameters	Value/Type
Learning rate ($\:\alpha\:)$	0.005
Hidden layer units	64
Batch size	32
Epochs	150
Activation function	Tanh
Dropout rate	0.3
Optimizer	Adam

**Fig. 5:**
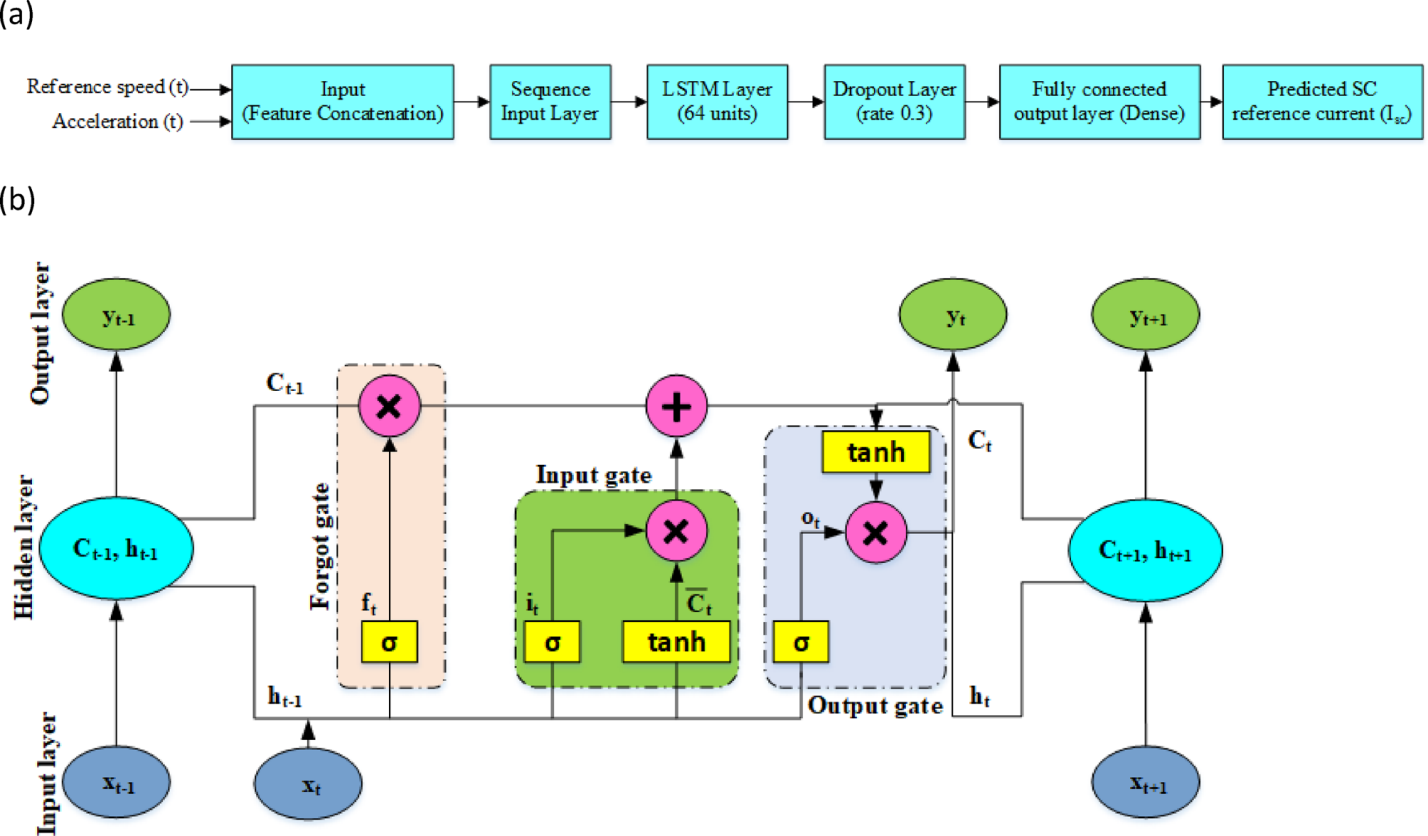
(**a**). LSTM model block diagram.(**b**). LSTM framework.

The LSTM-based model learns to map time-series inputs (acceleration and speed) to the desired output (SC current) using a memory-based architecture. As shown in Fig. 5(a), the network consists of an input concatenation stage, a sequence input layer, an LSTM layer (64 units), a dropout layer for regularization, and a fully connected output layer.

The core of this model is the LSTM layer, which can capture temporal dependencies in the input features through its internal memory mechanism. The structure of a single LSTM unit is illustrated in Fig. 5(b), and its internal operations are mathematically governed by the following equations:

19 $$\:{f}_{t}=\:\sigma\:({W}_{f}\left[{h}_{t-1},{x}_{t}\right]+{b}_{f}$$.

This is the forget gate ($\:{\varvec{f}}_{\varvec{t}}$), which determines how much of the previous memory ($\:{\varvec{C}}_{\varvec{t}-1}$) should be retained or discarded.

20 $$\:{i}_{t}=\:\sigma\:({W}_{i}\left[{h}_{t-1},{x}_{t}\right]+{b}_{i}$$.

The input gate controls how much new information from the current input $\:\left({x}_{t}\right)$ should be stored in the memory.

21 $$\:{\stackrel{-}{C}}_{t}=\text{tan}h({W}_{C}\left[{h}_{t-1},{x}_{t}\right]+{b}_{C}$$.

Here, the candidate cell state $\:({\stackrel{-}{C}}_{t}$) is generated as a potential update to the internal memory, using a $\:\text{tan}h$ activation to scale values between − 1 and 1.

22 $$\:{C}_{t}={f}_{t}{.C}_{t-1}+{i}_{t}.{\stackrel{-}{C}}_{t}$$.

This is the memory update equation, where the new cell state $\:{(C}_{t})$​ is computed by combining the retained memory and the new candidate update, weighted by the forget and input gates respectively.

23 $$\:{o}_{t}=\:\sigma\:({W}_{o}\left[{h}_{t-1},{x}_{t}\right]+{b}_{o}$$.

The output gate ($\:{o}_{t}$) decides which parts of the memory should influence the output at the current time step.

24 $$\:{h}_{t}=\:{o}_{t}.\text{tan}h\left({C}_{t}\right)$$.

Finally, the hidden state output ($\:{h}_{t}$)​ is computed based on the updated memory ($\:{C}_{t}$)​ and controlled by the output gate. Through this gated mechanism, the LSTM layer learns temporal dependencies in the sequence of inputs (reference acceleration and speed), enabling it to generate a time-varying prediction of the supercapacitor current with memory of past context.

**Fig. 6:**
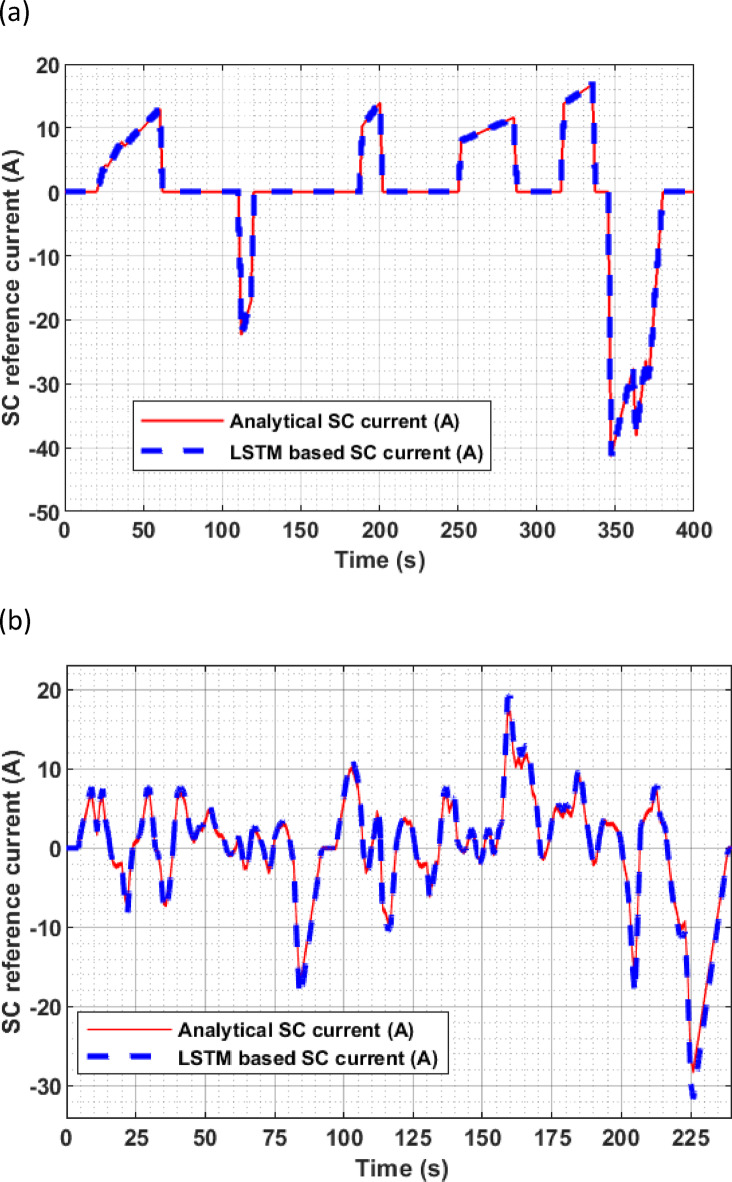
**(A)**Analytical vs LSTM based SC reference current under EUDC(**B**). Analytical vs LSTM based SC reference current under IM 240 drive cycle.

Figures6(a) and (b) compare the SC reference current obtained from the analytical model with the LSTM-based machine learning prediction for the EUDC and IM240 drive cycles, respectively. In both cases, the predicted current closely aligns with the analytical reference, effectively capturing key transients during acceleration and deceleration phases. Although minor differences may exist, particularly at rapid current transitions, these deviations are negligible and fall within acceptable error margins. This demonstrates that the LSTM model generalizes well across dynamic drive cycles, providing a reliable and data-driven approach for SC current prediction.

Quantitative error analysis for both drive cycles is summarized in Table 3 using four standard metrics: Root Mean Square Error (RMSE), Mean Absolute Error (MAE), Mean Absolute Percentage Error (MAPE), and Coefficient of Determination (R²)^69,70^. These are mathematically defined below and adapted to reflect the deviation between the predicted SC current from the LSTM model and the analytical reference current. The RMSE is calculated as given in Eqs. (25),

25 $$\:RMSE\:\left(A\right)=\:\sqrt{\frac{1}{n}\sum\:_{i=1}^{n}{\left({I}_{sc}\right(i)-{I}_{LSTM}(i\left)\right)}^{2}}$$.

where $\:{I}_{sc}\left(i\right)$ and $\:{I}_{LSTM}\left(i\right)$ are the analytical reference supercapacitor current and LSTM-predicted current at the $\:i-th$ time step, respectively, and $\:n$ is the total number of samples. The MAE measures the average magnitude of absolute error and is expressed in Eqs. (26),

26 $$\:MAE\:\left(A\right)=\:\frac{1}{n}\sum\:_{i=1}^{n}\left|{I}_{sc}\left(i\right)-{I}_{LSTM}\left(i\right)\right|\:$$.

The MAPE quantifies the error relative to the reference values in percentage form as given in Eqs. (27),

27 $$\:MAPE\:\left(\%\right)=\:\frac{100}{n}\sum\:_{i=1}^{n}\left|\frac{{I}_{sc}\left(i\right)-{I}_{LSTM}\left(i\right)}{{I}_{sc}\left(i\right)}\right|\:$$.

The coefficient of determination $\:{R}^{2}$ indicates how well the predicted current matches the reference current as shown in Eqs. (28),

28 $$\:{R}^{2}=1-\frac{\sum\:_{i=1}^{n}{\left({I}_{sc}\right(i)-{I}_{LSTM}(i\left)\right)}^{2}}{\sum\:_{i=1}^{n}{\left({I}_{sc}\right(i)-\stackrel{-}{{I}_{sc}})}^{2}}$$.

where $\:\stackrel{-}{{I}_{sc}}$ is the mean of the reference current. These metrics collectively evaluate prediction fidelity. Low RMSE, MAE, and MAPE values, combined with high $\:{R}^{2}$ values close to unity, confirm the effectiveness of the ONNX-based LSTM model in generating accurate SC current trajectories for hybrid EV systems.

**Table 3:** Error metrics for SC reference current prediction.

Metric	EUDC	IM240
RMSE (A)	1.26	1.13
MAE (A)	0.87	0.79
MAPE (%)	2.41	2.12
$\:{R}^{2}$	0.9376	0.9481

Figure 8 illustrates the Simulink implementation of supercapacitor reference current generation using a machine learning algorithm integrated via an ONNX Predict block. The reference driving cycle (EUDC – 400 s) provides time-series data for vehicle speed and acceleration, which are preprocessed and reshaped to form the input vector. This input is fed into the ONNX-based prediction block, which encapsulates a trained LSTM network developed in Python, enabling real-time prediction of the SC reference current. A closed-loop PI controller minimizes the error between the predicted reference and the actual SC current. The control output regulates the duty cycle of the PWM generator that controls the bidirectional DC-DC converter, enabling precise dynamic power flow between the battery and supercapacitor. This configuration ensures fast transient support and efficient energy management in the HESS.

**Fig. 7:**
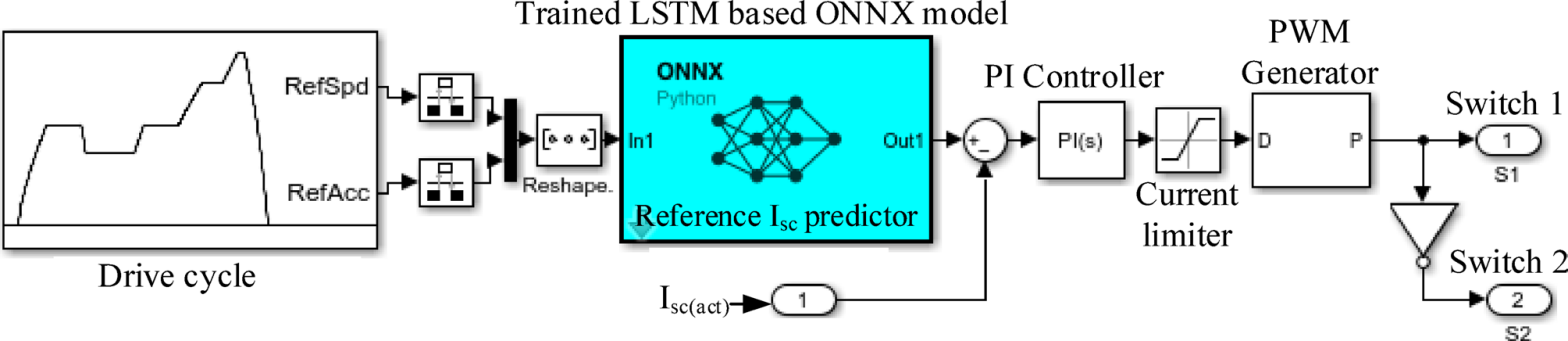
Supercapacitor reference current generation using a machine learning algorithm.

## Implementation of the proposed system

**Fig. 8:**
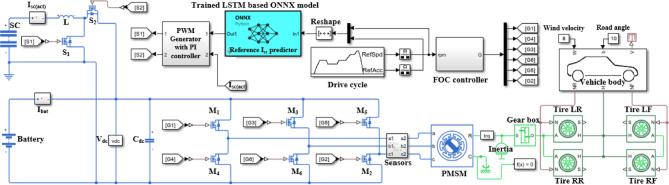
Simulink model of battery and supercapacitor energy management in PMSM based powertrain.

The MATLAB Simscape model illustrated in Fig. 8 represents the complete implementation of an electric vehicle powertrain based on the Nissan Sakura specifications, detailed in Table 4. The system architecture includes a lithium-ion battery, a supercapacitor, a bidirectional DC-DC converter, a three-phase voltage source inverter, and a 47-kW permanent magnet synchronous motor (PMSM), consistent with the actual drivetrain configuration of the vehicle.

**Table 4:** Technical specifications of power train model.

Parameters	Value	Unit	Parameters	Value	Unit
Rated traction motor power	47	$\:\text{k}\text{W}$	Length of the vehicle	3395	$\:\text{m}\text{m}$
Rated electromagnetic torque	195	Nm	Width of the vehicle	1475	$\:\text{m}\text{m}$
Rated battery capacity	20	$\:\text{k}\text{W}\text{h}$	Height of the vehicle	1655	$\:\text{m}\text{m}$
Battery nominal voltage	350	V	Base of the wheel	2495	$\:\text{m}\text{m}$
SC capacitance	32	F	Gross weight of the vehicle	1070	$\:\text{k}\text{g}$
Number of series SC	130	-	Maximum passenger load	400	$\:\text{k}\text{g}$
Number of parallel SC	10	-	Size of the wheel	155/65 R14 75 S	-
Dc link capacitance	1800	µF	Gear efficiency	0.8 to 0.9	-
Air density	1.2	$\:\text{k}\text{g}/{\text{m}}^{3}$	Coefficient of rotating mass	1.04	-
Ambient pressure	100	$\:\text{k}\text{P}\text{a}$	Coefficient of air drag	0.29	
Ambient temperature	298.2	K	Coefficient of rolling resistance	0.012	-
Rated speed of the vehicle	130	$\:\text{k}\text{m}/\text{h}\text{r}$	Recovery energy efficiency	0.65	-

A data-driven energy management strategy is employed using a machine learning-based ONNX-integrated LSTM model. This model dynamically predicts the supercapacitor reference current ($\:{I}_{sc})$ based on real-time drive cycle inputs—namely, vehicle speed and acceleration. The predicted reference is utilized to regulate power exchange between the battery and SC, ensuring efficient transient power support during dynamic conditions such as acceleration and braking.

The bidirectional DC-DC converter, positioned between the SC and the DC-link, plays a key role in controlling the direction and magnitude of energy flow. It consists of two controllable switches ($\:{S}_{1}\:$and $\:{S}_{2}$) and a coupling inductor (L). During vehicle acceleration, the LSTM model outputs a positive $\:{I}_{sc}$, prompting the PI-controlled PWM generator to activate switch $\:{S}_{1}$. This allows the supercapacitor to discharge through inductor L, delivering supplementary power to the DC-link. In this scenario, the inductor operates in boost mode, stepping up the SC voltage to the DC-link level, thereby helping the battery meet peak power demands.

Conversely, during deceleration or regenerative braking, the PMSM feeds energy back through the inverter. The controller activates switch $\:{S}_{2}$, and the DC-DC converter operates in buck mode and charges the SC. This operation enhances braking energy recovery and overall energy utilization. During steady-state cruising, when power demand is low and nearly constant, the SC remains inactive, and the battery alone provides the required propulsion power.

To ensure stable system operation, a DC-link capacitor ($\:{C}_{dc}$) is connected across the inverter input to mitigate voltage ripples and absorb instantaneous transients. Real-time feedback of the actual SC current ($\:{I}_{sc\left(act\right)}$) is compared with the predicted reference ($\:{I}_{sc}$) to enable closed-loop regulation via the PI controller.

The model also includes field-oriented control (FOC) of the PMSM for high-performance torque and speed regulation. A gearbox transmits the motor torque to the drivetrain, with vehicle dynamics simulated using the EUDC and the IM240 standard for evaluating energy flow, regenerative efficiency, and range under various conditions.

This comprehensive simulation framework captures the real-world behavior of the Nissan Sakura EV and validates the effectiveness of the proposed LSTM-based energy management strategy. It provides a foundation for further refinement of power distribution under acceleration, deceleration, and cruising states.

## Results and discussion

### Comparative performance analysis of BEV and HBEV under the EUDC drive cycle

**Fig. 9:**
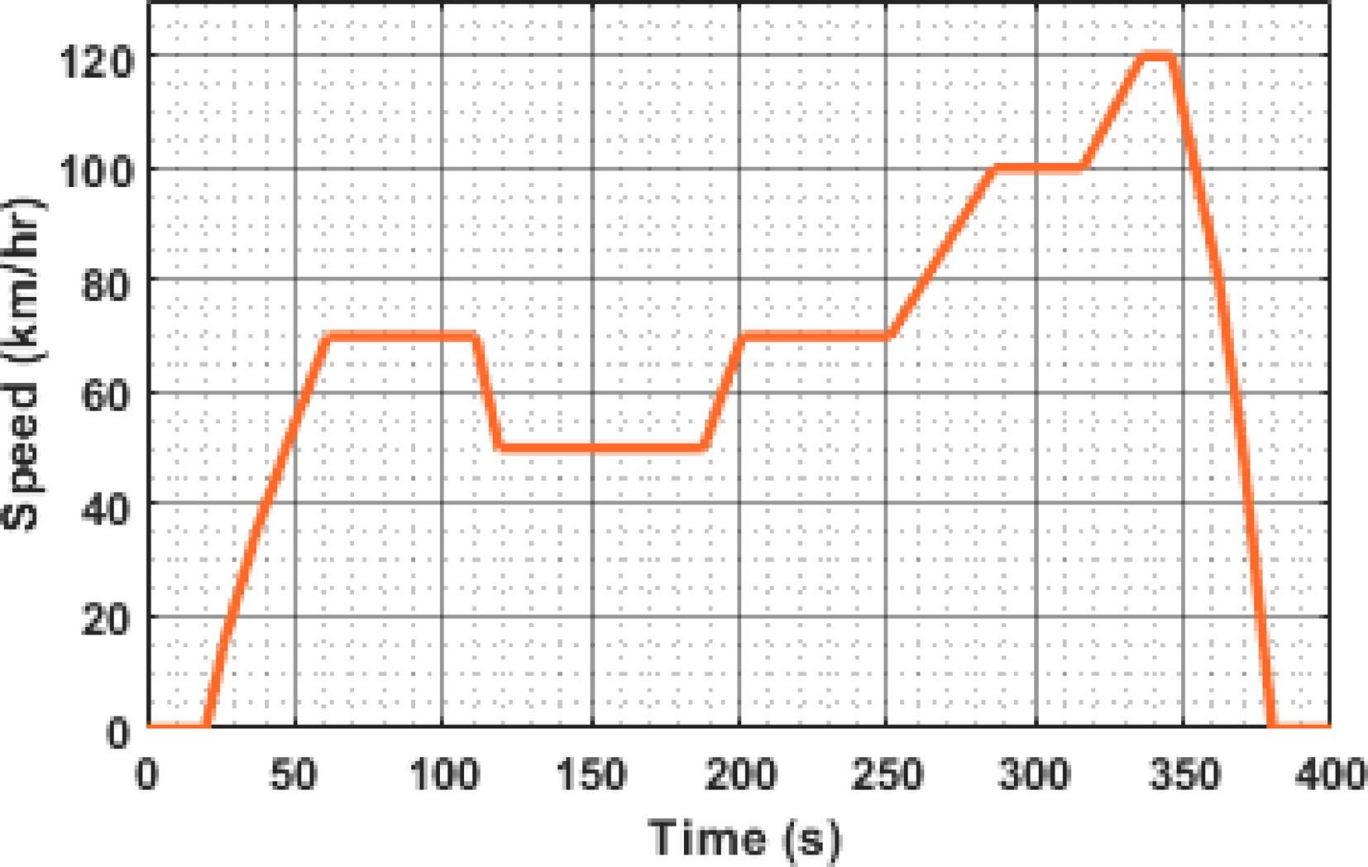
EUDC drive cycle.

The European Urban Driving Cycle (EUDC), as shown in Fig. 9, serves as a representative profile for evaluating the dynamic performance of EV powertrains under mixed urban and highway conditions. The profile features multiple speed transitions, including sharp accelerations and frequent decelerations, simulating realistic scenarios such as stop-and-go traffic and highway cruising.

The cycle begins with an initial acceleration phase, where the vehicle speed ramps up from 0 km/h to approximately 70 km/h at around 60 s. A brief cruising phase follows, after which another acceleration peak occurs at approximately 290 s, reaching 100 km/h. The highest speed is observed near 120 km/h just before 400 s, followed by a rapid deceleration to zero.

Such speed variations demand agile energy management. During acceleration phases, the SC is expected to mitigate transient power demands, thereby reducing battery peak current and improving response time. Conversely, during deceleration, regenerative braking allows energy to be recovered and partially stored in the SC. This dual role of the SC plays a pivotal part in improving battery life, reducing energy consumption, and enhancing thermal performance.

The following subsections evaluate the system’s response—particularly the SC current, battery dynamics, and traction motor behavior—under the EUDC profile to assess the effectiveness of the proposed ONNX-deployed LSTM-based current prediction framework.

**Fig. 10:**
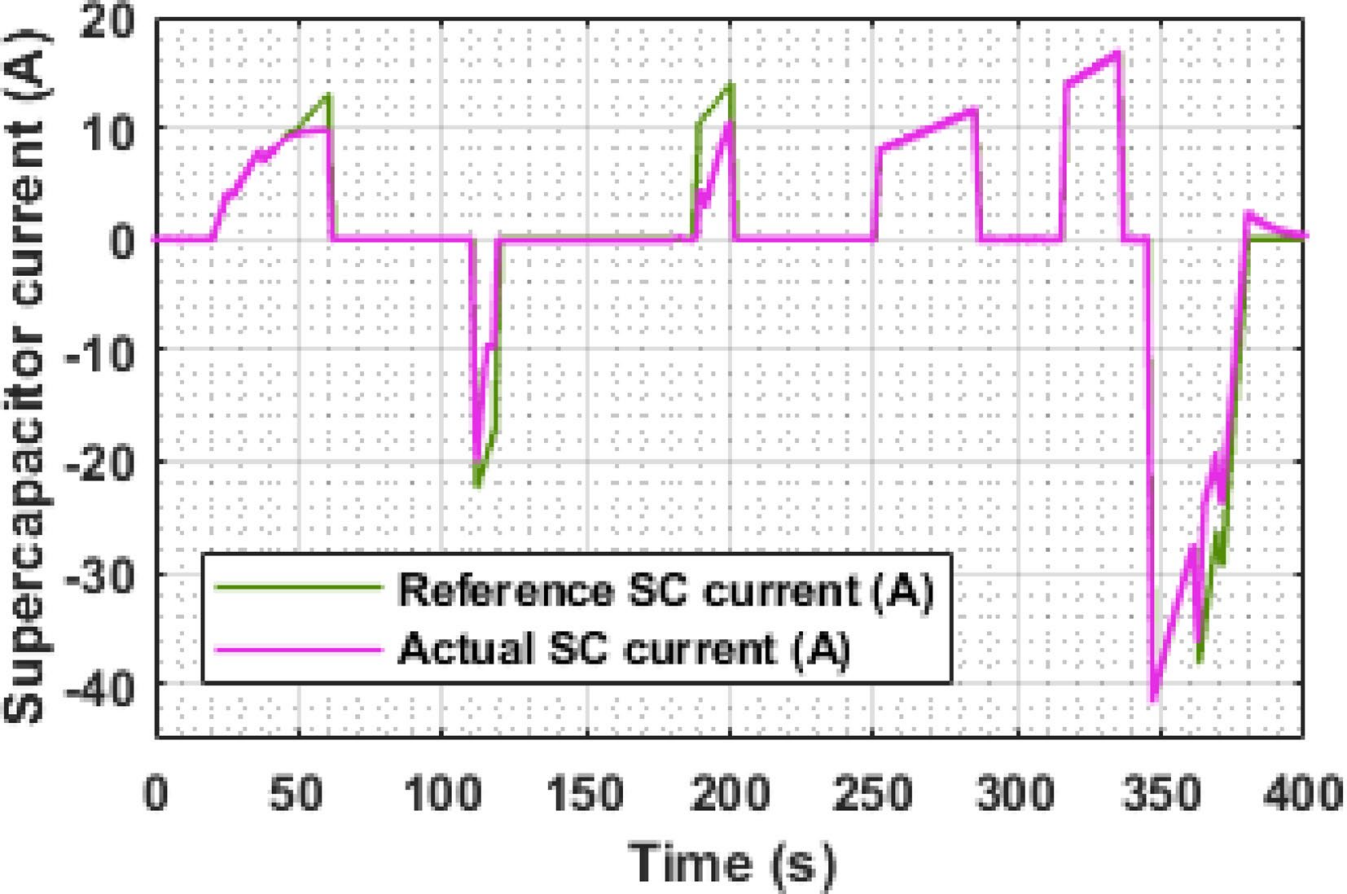
Reference and actual current of SC.

Figure 10 illustrates the reference and actual SC current profiles under the EUDC cycle. The reference current is generated via the ONNX-deployed LSTM model to represent the ideal assistance expected from the SC, while the actual current reflects its dynamic response through the bidirectional converter.

During high acceleration phases (e.g., t = 60 s, 200 s, 284 s, and 344 s), the SC actively discharges, supporting peak load demands by delivering 10 A, 10.5 A, 11.8 A, and 16.6 A, respectively. These discharges directly correspond to speed ramps identified in Fig. 9, confirming the SC’s role in reducing instantaneous battery current stress and enhancing system responsiveness.

In contrast, during regenerative braking at t = 122 s and 347 s, the SC absorbs energy with peak currents of − 20 A and − 41.5 A, respectively. The close tracking between the reference and actual current in these intervals indicates effective energy capture and bidirectional power control.

During cruising intervals, the SC remains inactive, and the battery sustains the load entirely. These periods highlight the model’s adaptive behavior, ensuring SC engagement only during high dynamic transients, thus contributing to overall energy optimization.

**Fig. 11:**
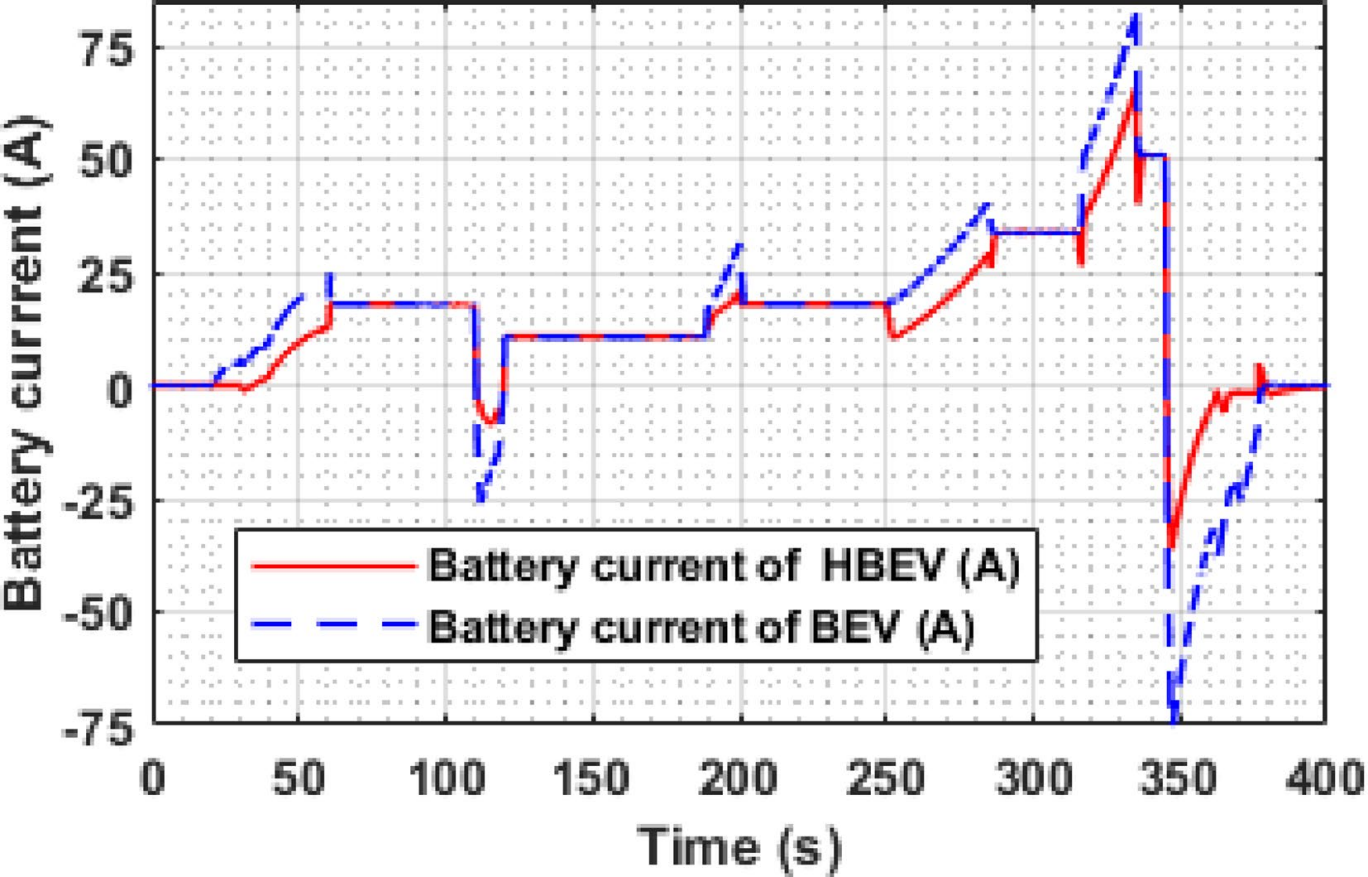
Battery current of BEV and HBEV.

The battery current profiles for both BEV and HBEV under the EUDC drive cycle are compared in Fig. 11. During acceleration intervals (e.g., at 60 s, 200 s, 284 s, and 344 s), the BEV battery current peaks sharply at 25 A, 31 A, 41 A, and 82.7 A, respectively. In contrast, the HBEV battery current is significantly lower at 18 A, 20.3 A, 29.2 A, and 65.1 A for the same events—corresponding to peak current reductions of approximately 28%, 34%, 28.8%, and 21.3%. This indicates that the SC effectively alleviates transient load demand, mitigating battery stress during high-power acceleration.

During regenerative braking periods (notably around 122 s and 347 s), the BEV configuration registers deep negative spikes of −26 A and − 75 A, reflecting direct regenerative energy recovery into the battery. In comparison, the HBEV profile is more moderate, with corresponding values of −3.3 A and − 36 A. These reductions confirm that the SC absorbs a considerable share of braking energy, avoiding harsh reverse current flow into the battery.

For cruising intervals—such as between 61 and 110 s, 120–186 s, and other marked regions—the current profiles for both systems converge, indicating that the SC remains inactive and the battery alone meets the propulsion demand.

In addition to peak current suppression, the RMS battery current for the EUDC cycle is also reduced due to SC integration. Specifically, the BEV registers an RMS current of 19.82 A, while the HBEV records 19.24 A—yielding a net reduction of approximately 2.93%. This modest but measurable decrease reflects the overall smoothing effect of the SC across varying drive conditions. Consequently, the HESS configuration substantially improves current uniformity, reduces thermal loading, and supports extended battery durability compared to conventional BEV operation.

**Fig. 12:**
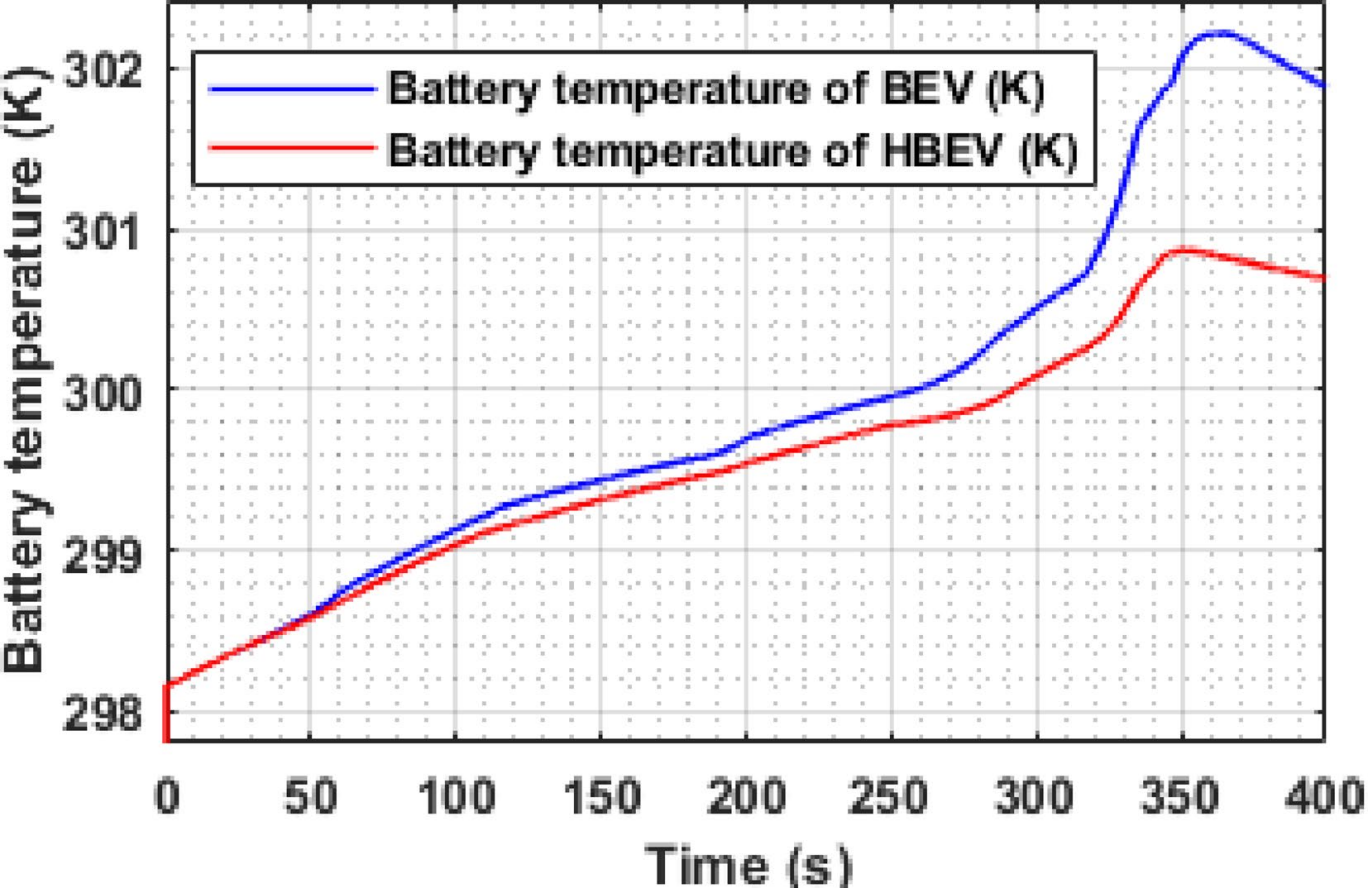
Battery temperature of BEV and HBEV.

Battery temperature evolution for both BEV and HBEV configurations under the EUDC drive cycle is examined in Fig. 12. The thermal response is obtained using a MATLAB Simulink-based battery thermal model that incorporates a 50–50% ethylene glycol cooling system. A steeper rise in BEV battery temperature is observed, especially beyond 300 s, coinciding with high-power acceleration and regenerative braking phases. The inclusion of the SC in the HBEV mitigates these thermal spikes by buffering peak current exchanges.

At approximately 360 s, the BEV battery reaches a peak temperature of 302.2 K, primarily driven by a high regenerative current of −75 A. In contrast, the HBEV maintains a lower peak of 300.8 K under similar load conditions, where the peak charging current is reduced to −36 A. Over the entire cycle, average battery temperatures for BEV and HBEV are calculated as 300.5 K and 299.1 K, respectively. While the ~ 1.4 K difference may seem modest, it plays a crucial role in minimizing thermal degradation, enhancing battery reliability, and reducing the risk of thermal runaway during sustained operation.

The observed disparity in temperature rise is directly linked to the current behavior outlined in Fig. 11, wherein the BEV experiences larger current excursions, resulting in higher I²R losses. The HBEV, on the other hand, benefits from SC support that reduces thermal stress by smoothing power flow and moderating internal heating within the battery pack.

**Fig. 13:**
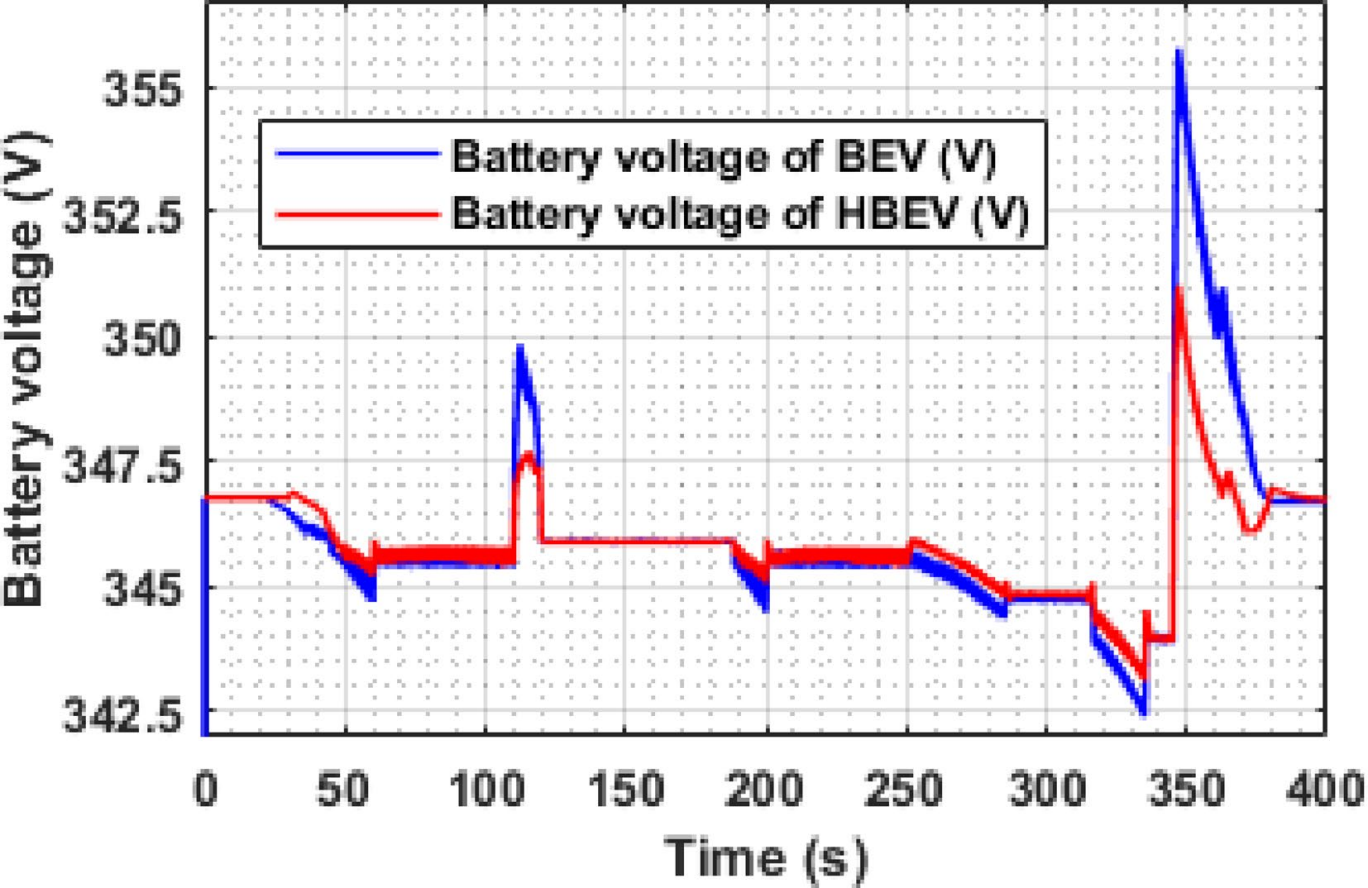
Battery voltage of BEV and HBEV.

Battery voltage profiles for both BEV and HBEV configurations under the EUDC drive cycle are analyzed in Fig. 13. The voltage response is governed by the discharge behavior during acceleration and the regenerative charging during deceleration phases. Due to higher transient loads, BEV experiences more pronounced voltage drops, while the HBEV benefits from SC support, which moderates these fluctuations.

At major acceleration points (t ≈ 60 s, 200 s, 284 s, and 344 s), the BEV battery voltage drops from its nominal 346.7 V to 344.7 V, 344.5 V, 344.4 V, and 342.4 V, respectively. In contrast, the corresponding voltage drops in the HBEV are mitigated, registering higher values of 345.1 V, 345.0 V, 344.81 V, and 343.1 V. This demonstrates the SC’s role in alleviating transient loading on the battery during power-intensive events.

During regenerative braking, particularly at t ≈ 347 s, the BEV battery voltage peaks at 355.8 V, while the HBEV reaches 351.0 V. Over the entire drive cycle, average battery voltages are calculated as 346.0 V for the BEV and 345.9 V for the HBEV. Based on the maximum and minimum observed values, the voltage ripple is quantified as 3.87% for the BEV and 2.28% for the HBEV, indicating a 1.59% reduction in the HBEV due to supercapacitor buffering.

The HESS configuration ensures a more stable voltage profile by moderating the impact of rapid load changes. This not only enhances voltage regulation but also contributes to reduced battery stress, improved conversion efficiency, and more consistent vehicle performance—particularly during high dynamic load phases such as acceleration and braking. The data clearly illustrate the stabilizing influence of SC support on battery voltage behavior.

**Fig. 14:**
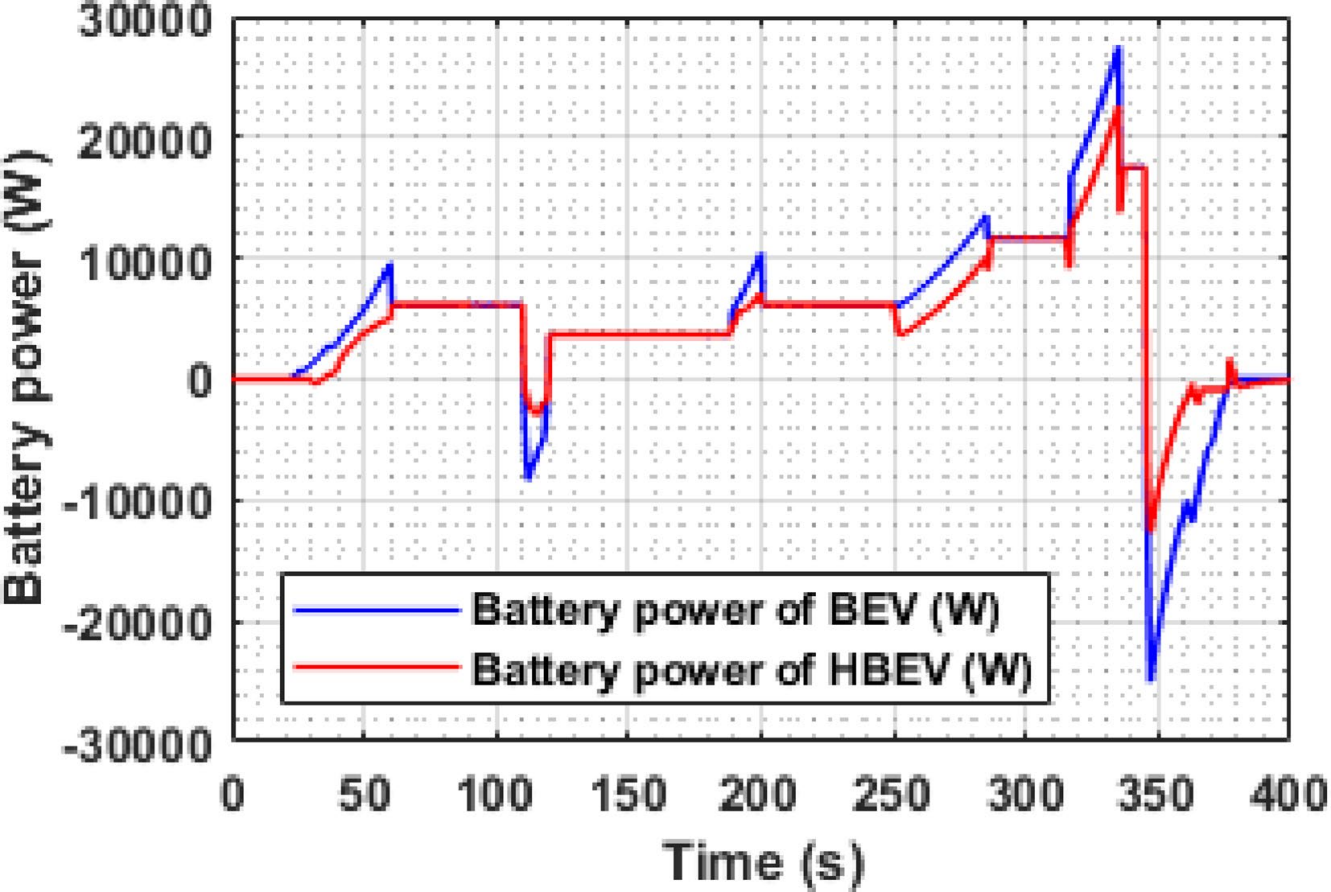
Battery power of BEV and HBEV.

Figure 14 presents the battery power response of BEV and HBEV configurations throughout the EUDC cycle. Power demand is directly influenced by vehicle acceleration and deceleration, with sharp transitions causing significant stress on the battery in conventional BEVs.

During peak acceleration at t ≈ 334.7 s, the BEV records a battery power demand of 27.92 kW, while the HBEV limits it to 22.49 kW, reflecting a reduction of 18.1%. This mitigation is attributed to the supercapacitor, which supplements the traction motor during transients, offloading the battery.

Similarly, during regenerative braking at t ≈ 347 s, the BEV absorbs a peak negative power of − 25.1 kW, whereas the HBEV restricts it to − 12.7 kW, resulting in a 49.4% reduction in peak charging power. This reduced regenerative burden indicates that the SC captures a substantial portion of the recovered energy, thereby protecting the battery from high current influx. Overall, the HBEV configuration demonstrates significantly smoother power transitions, reducing peak power excursions on both ends. The presence of the SC thus proves vital in achieving a more balanced and robust energy management framework under dynamic driving conditions.

**Fig. 15:**
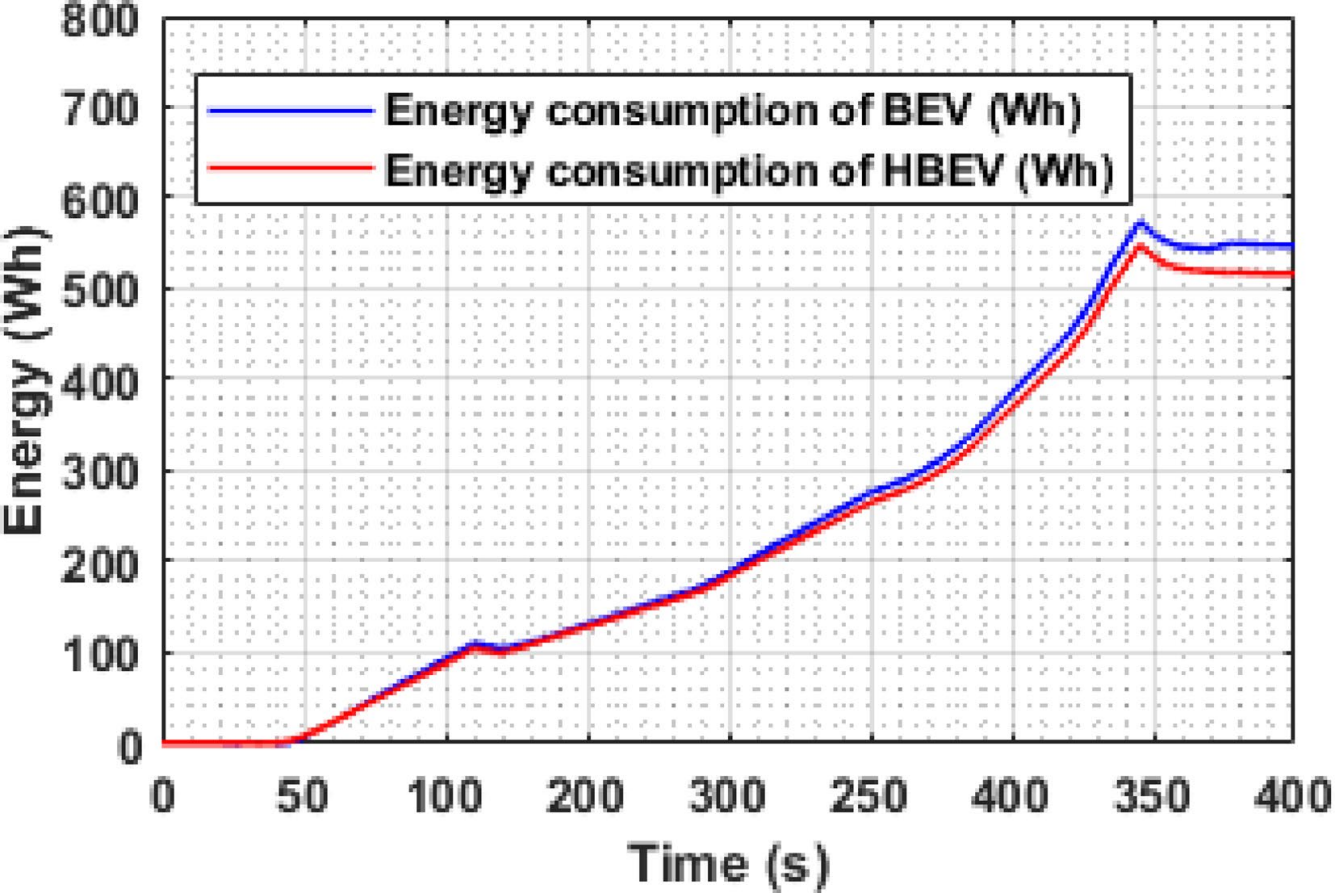
Battery energy consumption of BEV and HBEV..

The energy consumption trajectories of both BEV and HBEV are shown in Fig. 15. Both systems exhibit a steady rise in energy usage throughout the EUDC drive cycle, with a sharper slope observed during acceleration-intensive intervals. Following the full completion of the cycle, the total energy consumed by the BEV reaches 547.5 Wh, whereas the HBEV system reports a lower value of 516 Wh, reflecting a net reduction of 31.5 Wh, equivalent to 5.75%.

This decrease is primarily attributed to the SC’s role in handling transient power demands and regenerative absorption, which alleviates direct battery load. By reducing the battery’s active participation during dynamic transitions, the system improves overall energy efficiency across the drive cycle without compromising performance.

**Fig. 16:**
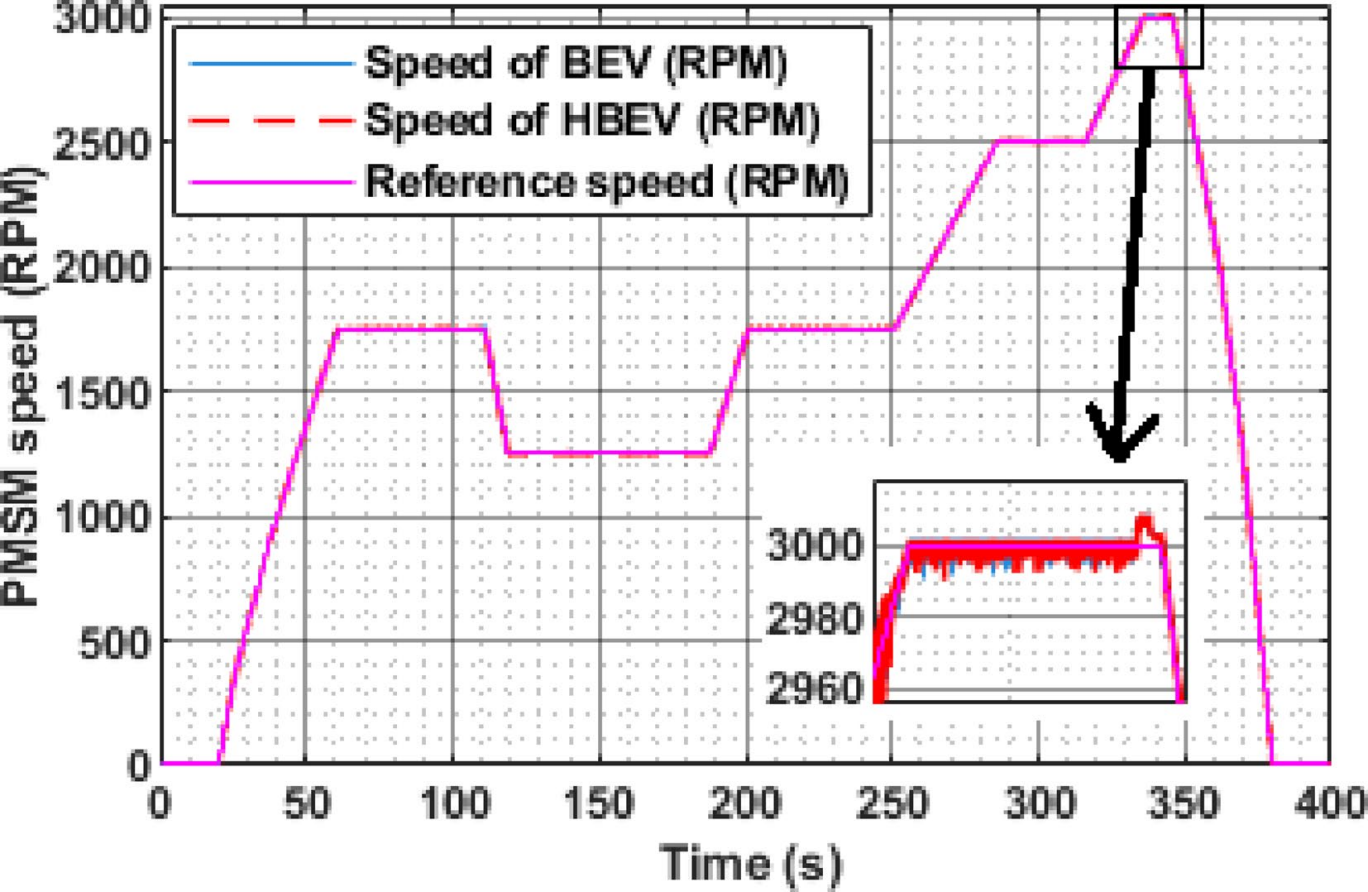
Reference and actual speed of PMSM.

The speed responses of both BEV and HBEV configurations are compared in Fig. 16. During constant-speed segments of the EUDC cycle—specifically over the intervals 61–110 s, 120–186 s, 201–250 s, 287–315 s, and 337–345 s—the observed motor speeds stabilize at approximately 1750 RPM, 1250 RPM, 1750 RPM, 2500 RPM, and 3000 RPM, respectively. In both systems, the PMSM effectively tracks the reference speed profile with high fidelity.

Minor deviations are seen during rapid transitions, largely attributed to inertia and transient response delays. Nonetheless, the accurate alignment between actual and reference speeds across both systems validates the effectiveness of the FOC strategy and confirms that the integration of SC assistance does not adversely impact dynamic speed control.

**Fig. 17:**
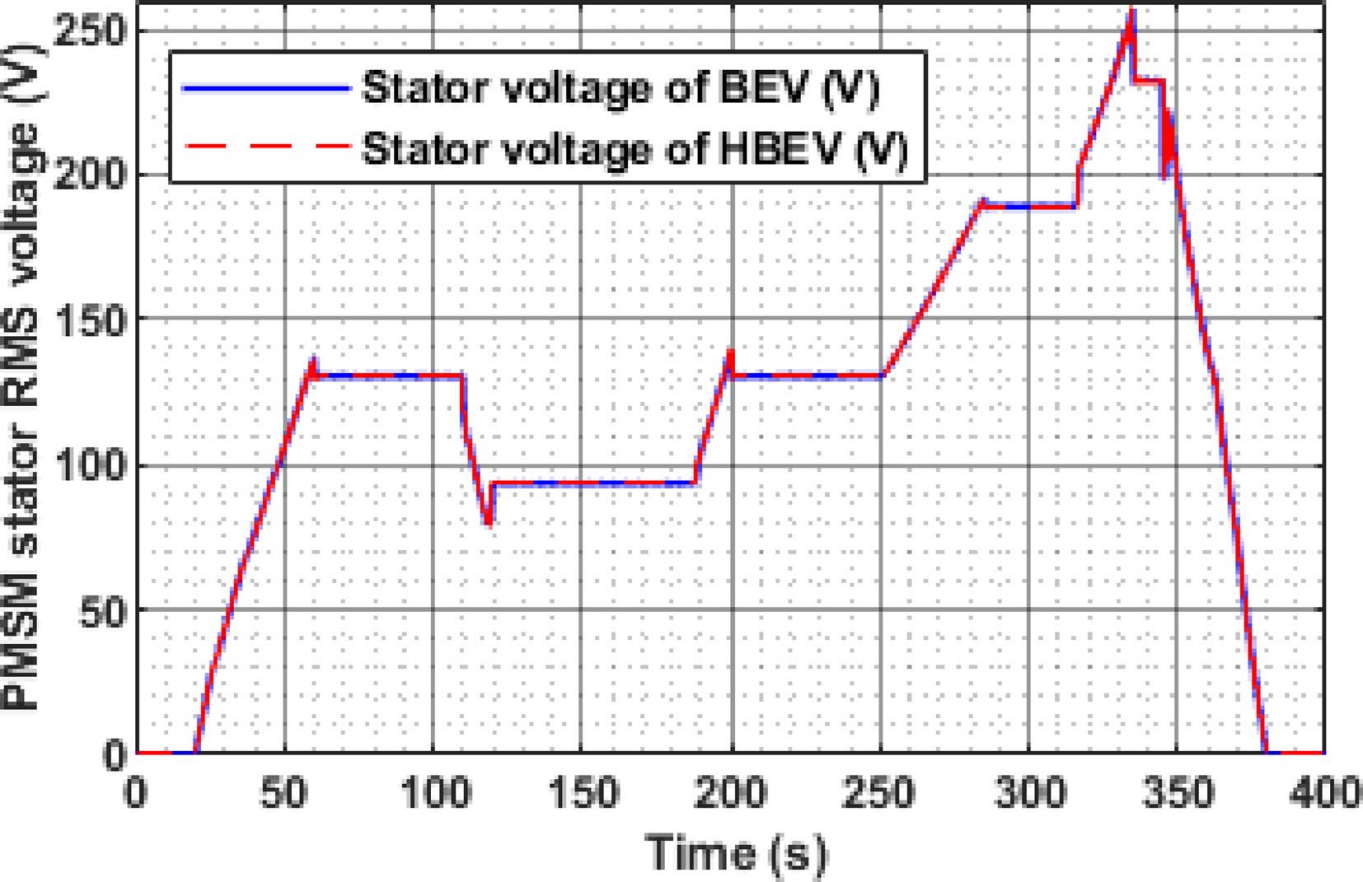
Stator voltage of PMSM.

Figure 17 presents the RMS stator voltage behavior of the PMSM for both BEV and HBEV configurations during the EUDC cycle. The waveform displays a step-like pattern that mirrors the speed transitions across the drive cycle. At rest (t < 50 s), the voltage remains at zero, indicating motor inactivity. As the cycle progresses, the stator voltage increases or decreases in response to the speed command.

For the steady-state speed intervals of 1750 RPM, 1250 RPM, 1750 RPM, 2500 RPM, and 3000 RPM, the corresponding stator voltages are observed to be 130.6 V, 93.5 V, 130.6 V, 189 V, and 232 V, respectively. These results confirm that the inverter appropriately adjusts the stator voltage to match the torque and speed requirements. The near-identical voltage profiles for BEV and HBEV also indicate that SC integration primarily affects current and power dynamics, without significantly altering the voltage regulation process.

**Fig. 18:**
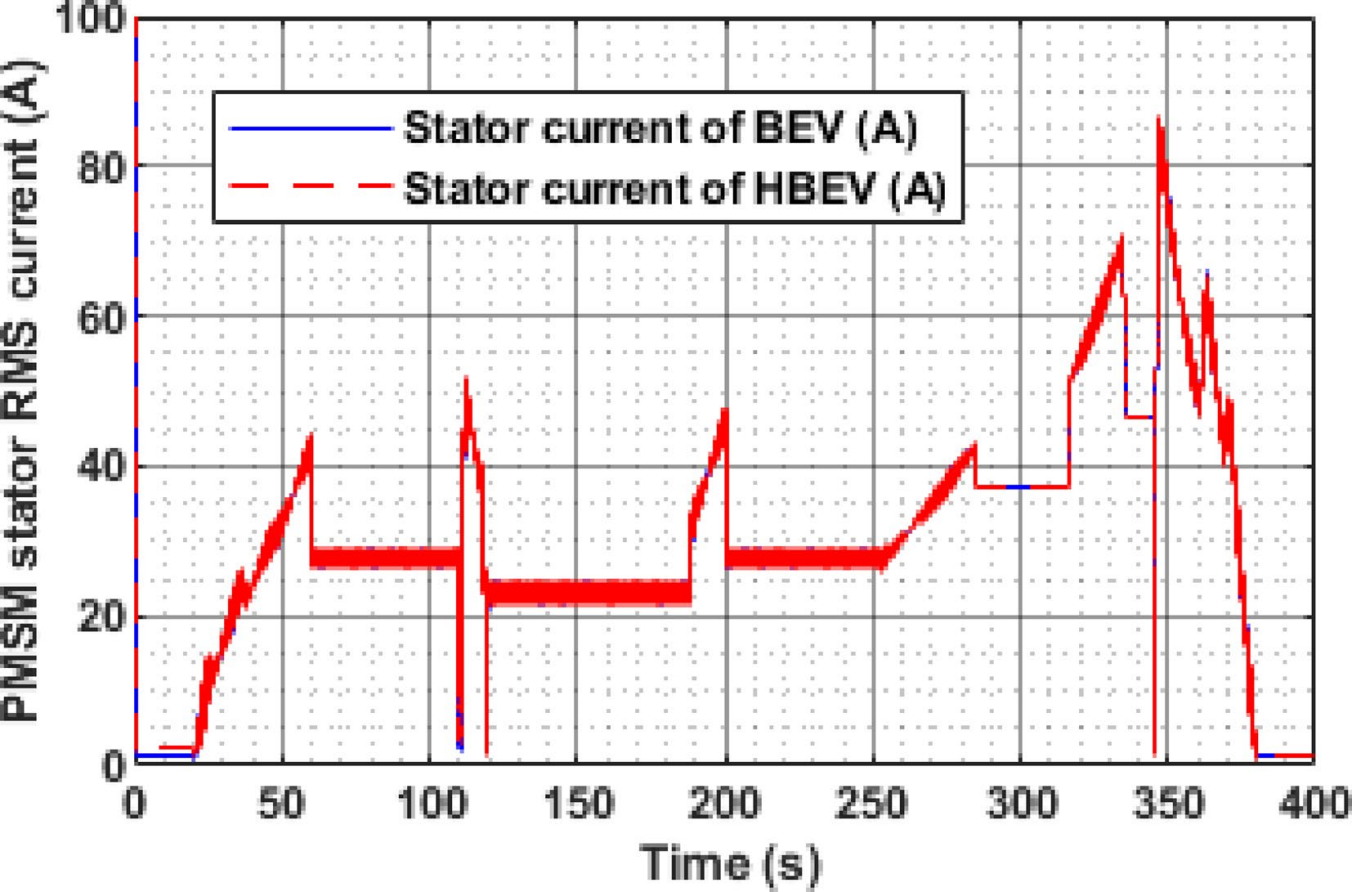
Stator current of PMSM.

The PMSM stator RMS current varies significantly in response to the dynamic phases of the EUDC cycle, as shown in Fig. 18. During acceleration peaks at approximately 60 s, 200 s, 284 s, and 344 s, the current rises to 44.4 A, 47.8 A, 43.2 A, and 71.1 A, respectively. Peak regenerative braking at 122 s and 347 s results in current spikes of 51.72 A and 86.84 A. In contrast, during constant-speed intervals, the current magnitude reduces substantially due to lower torque demand, improving overall system efficiency and reducing unnecessary energy losses.

**Fig. 19:**
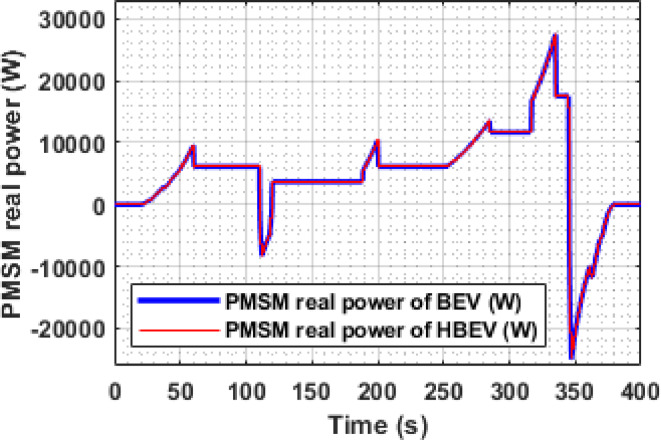
. Real power of PMSM.

The real power analysis of the PMSM during the EUDC cycle reveals distinct peaks during acceleration phases, with motor power reaching 9.5 kW, 10.32 kW, 13.6 kW, and 27.5 kW at approximately t = 60 s, 200 s, 284 s, and 344 s, respectively. These values align closely with the battery-side power demand of 9.64 kW, 10.47 kW, 13.8 kW, and 27.92 kW in the BEV, as shown in Fig. 19, from which an average inverter efficiency of 98.5% is inferred. During deceleration at t = 122 s and 347 s, the motor exhibits regenerative power peaks of −8.24 kW and − 25.1 kW, respectively, confirming effective energy recovery during braking.

**Fig. 20:**
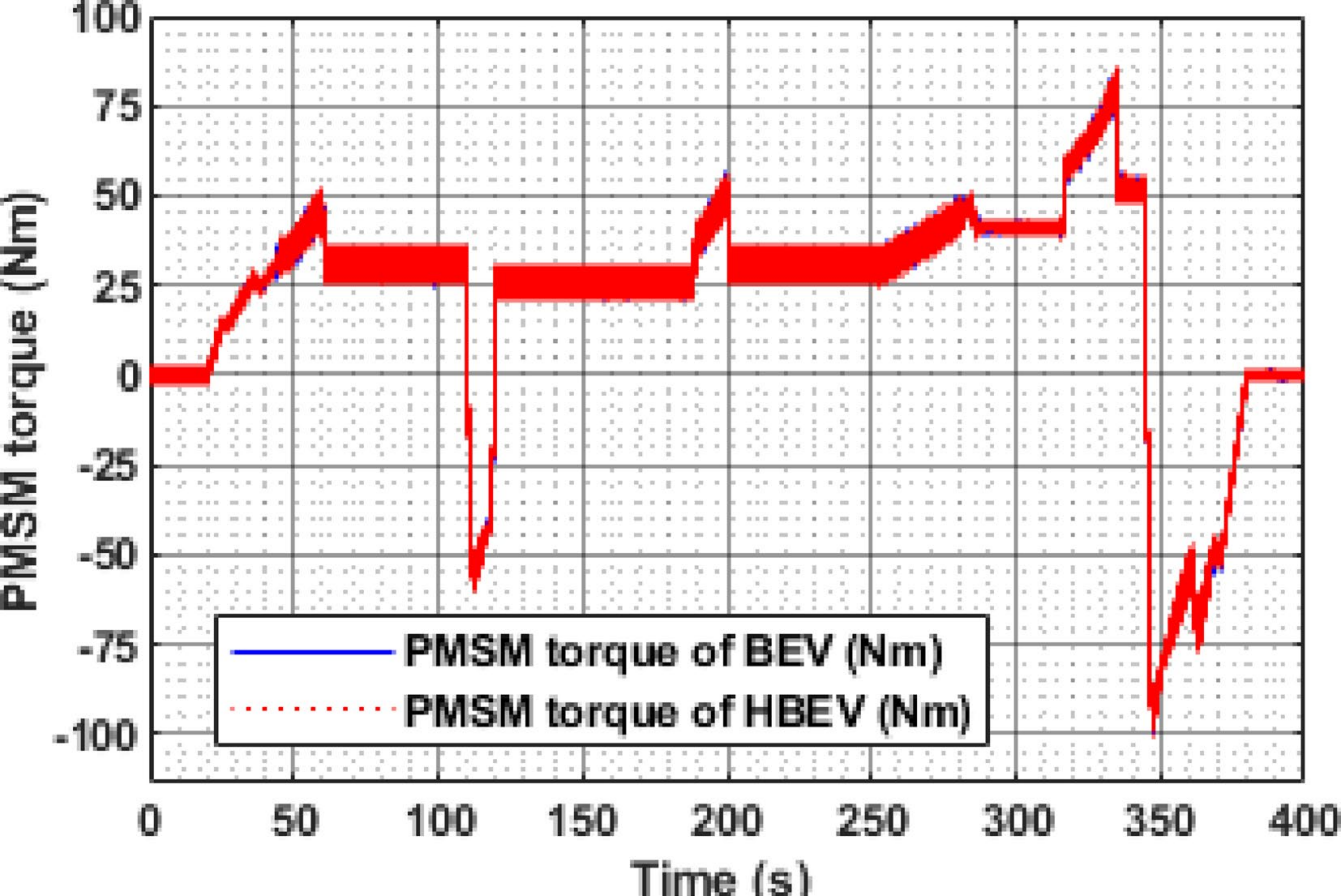
Electromagnetic torque of PMSM.

The electromagnetic torque developed by the PMSM varies according to load conditions in the EUDC cycle as indicated in Fig. 20. During acceleration phases at t ≈ 60 s, 200 s, 284 s, and 344 s, the motor produces peak torques of 52.26 Nm, 56 Nm, 50.8 Nm, and 85.1 Nm respectively, to overcome inertia and propel the vehicle. Conversely, during regenerative braking phases at t = 122 s and 347 s, the torque reverses direction, reaching peak negative values of −60.3 Nm and − 99.9 Nm, respectively, enabling effective deceleration and energy recovery.

### Comparative performance analysis of BEV and HBEV under the IM240 drive cycle

**Fig. 21:**
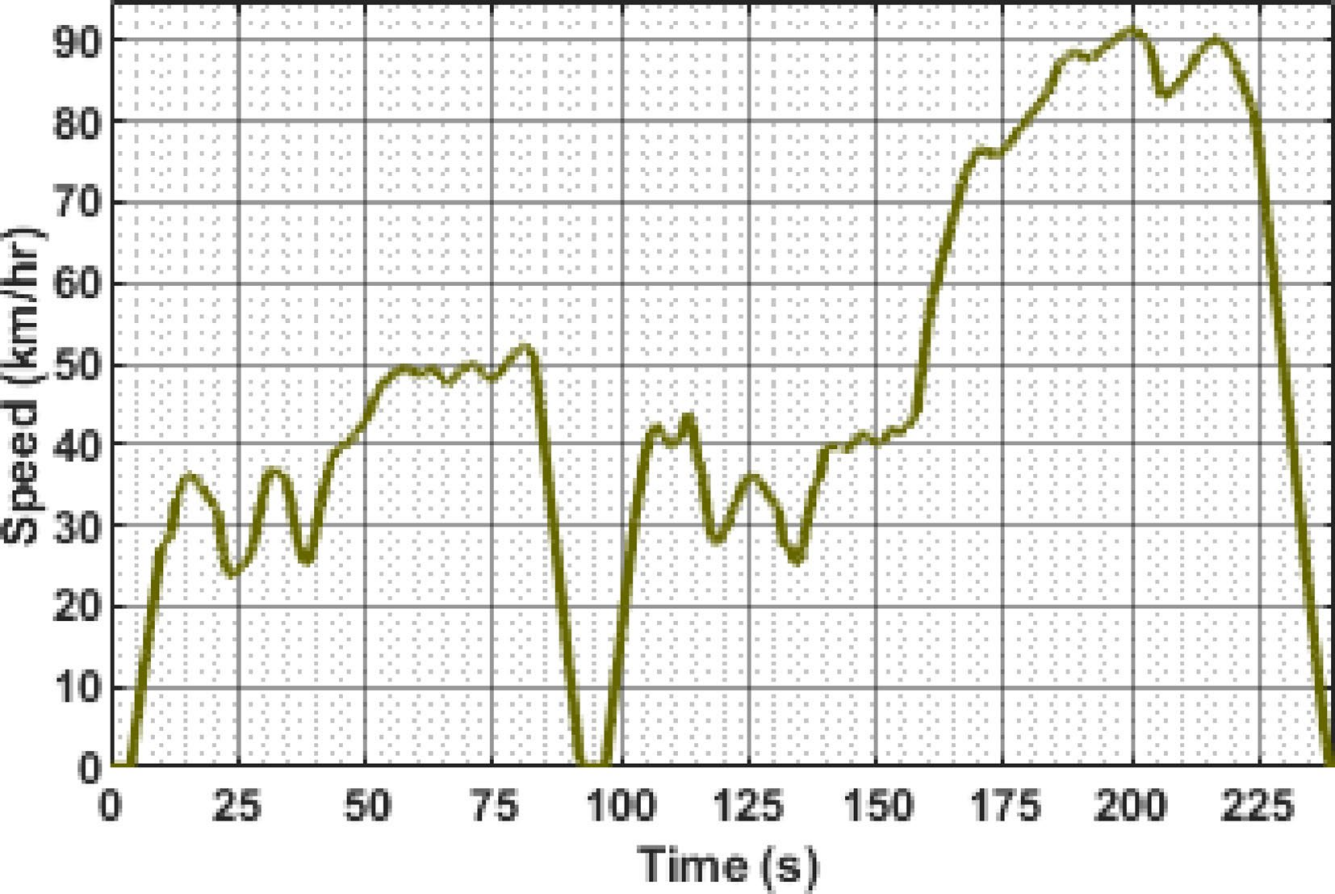
IM240 drive cycle.

The IM240 drive cycle features a dynamic velocity profile that includes repeated acceleration and deceleration events representative of urban and highway driving. As depicted in Fig. 21, the cycle begins from standstill, accelerating to around 36.5 km/h at 15.4 s, followed by multiple speed fluctuations. Notably, a sharp deceleration to 0 km/h occurs at 92.8 s, after which the vehicle accelerates rapidly to approximately 42.3 km/h by 107 s. The peak speed of 91.2 km/h is recorded at 200 s, culminating in a final rapid deceleration to zero. These dynamic variations significantly influence the loading patterns on the electric powertrain, directly affecting battery utilization, regenerative braking potential, and overall energy efficiency for both BEV and HBEV systems.

**Fig. 22:**
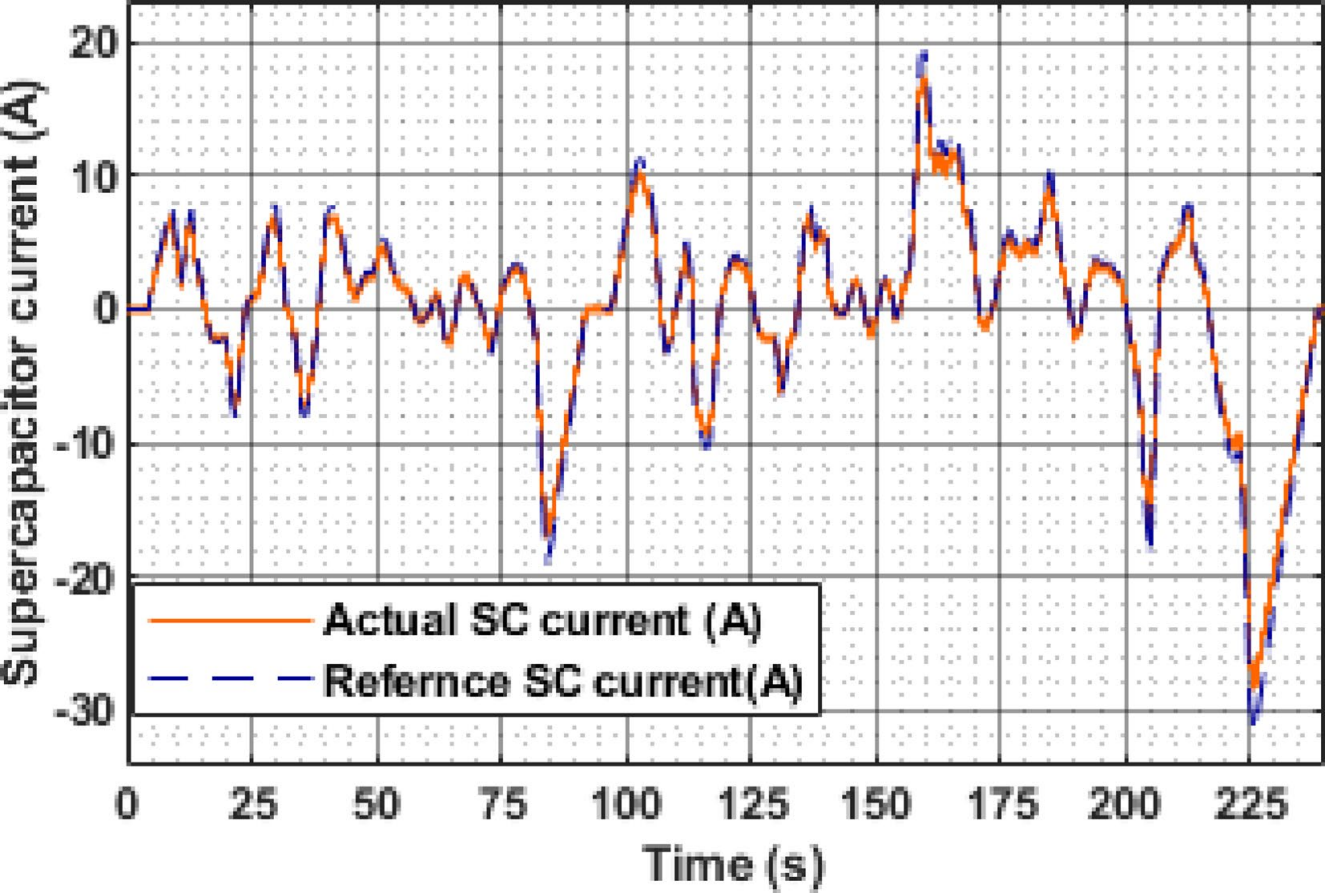
Reference and actual current of SC.

During the IM240 drive cycle, the reference and actual supercapacitor currents exhibit a closely matched profile, validating the performance of the SC control scheme. As shown in Fig. 22, the SC supplies 7.1 A at 9.1 s during the initial acceleration phase. A sharp deceleration at 83.7 s causes the SC to absorb regenerative energy, drawing a peak charging current of −16.8 A. The highest discharge current of 17.3 A is observed at 160 s during a strong acceleration, while the maximum regenerative current reaches − 27.56 A at 226 s. The minimal deviation between the reference (dashed blue) and actual (solid orange) current profiles throughout the cycle indicates accurate tracking and robust control, enabling efficient transient power handling in the hybrid system.

A clear difference in battery current profiles between BEV and HBEV configurations can be observed in Fig. 17, particularly during transient conditions. At 9.1 s, the BEV draws a peak current of 9.2 A during acceleration, whereas the HBEV current is limited to just 2.1 A—demonstrating a 77.2% reduction due to SC assistance. The highest acceleration demand occurs at 160 s, where the BEV reaches 51.59 A, while the HBEV peaks at 34.3 A, showing a 33.5% mitigation in current stress. During deceleration at 226 s, the BEV exhibits a regenerative peak of −62.66 A, while the HBEV reduces this to −35.1 A, yielding a 43.98% reduction in reverse current. These results demonstrate the SC’s effective role in moderating current surges during dynamic load events.

In addition to peak current mitigation, the RMS battery current across the IM240 cycle is also reduced. The BEV records an RMS current of 9.44 A, whereas the HBEV achieves a lower value of 8.83 A—reflecting a 6.46% overall reduction. This decrease underscores the SC’s contribution to smoothing battery load over time, reducing thermal cycling, and enhancing system efficiency under realistic urban driving scenarios.

The instantaneous temperature response of the battery, measured under a liquid cooling setup using ethylene glycol, is compared for BEV and HBEV configurations in Fig. 24. At 160 s, a peak battery current of 51.59 A in the BEV leads to a temperature rise reaching 300.1 K by 169 s, whereas the HBEV, limited to 34.3 A, peaks at only 299.3 K—a reduction of 0.8 K. Similarly, at 226 s during strong regenerative braking, the BEV temperature climbs to 301.43 K at 232 s, while the HBEV records 299.62 K, resulting in a more pronounced difference of 1.81 K.

By the end of the cycle, the final temperatures are 301.4 K for the BEV and 299.64 K for the HBEV, and the average battery temperatures across the entire cycle are 300.1 K and 298.2 K, respectively. This 1.9 K average reduction in the HBEV case highlights the supercapacitor’s role in suppressing temperature rise during current surges, contributing to more efficient thermal regulation under dynamic load conditions.

The instantaneous voltage response of the battery during the IM240 cycle is compared between BEV and HBEV configurations in Fig. 25. During acceleration phases, both systems exhibit voltage drops due to elevated current demands, with the most significant dip observed at 160 s, where the BEV voltage falls from 346.7 V to 343.4 V, while the HBEV exhibits a milder drop to 344.1 V. This reduced voltage sag in the HBEV highlights the stabilizing influence of the SC during transients.

Regenerative braking events result in voltage recovery, peaking around 225 s at 354.4 V in the BEV and 351 V in the HBEV. By absorbing part of the regenerative energy, the SC prevents sharp voltage overshoots in the hybrid configuration. Over the full cycle, average battery voltages are 346.9 V for the BEV and 346.7 V for the HBEV. Corresponding voltage ripple values are 3.17% and 1.99%, confirming a 1.18% reduction in the HBEV system. This smoother voltage profile reflects improved DC bus stability, contributing to reduced battery stress and enhanced converter performance.

The instantaneous battery power response during the IM240 cycle is compared for BEV and HBEV configurations in Fig. 26. At 160 s, corresponding to peak acceleration, the battery in the BEV supplies 17.1 kW, while in the HBEV, the demand drops to 11.7 kW. This 5.4 kW difference, amounting to a 31.6% reduction, is supplied by the SC, thereby offloading transient load from the battery.

During regenerative braking at 225 s, the BEV exhibits a peak negative power of −20.6 kW, compared to −12.44 kW in the HBEV, indicating that the SC absorbs 8.16 kW, reducing the battery’s charging stress by 39.6%. These results confirm the SC’s dual role, supporting propulsion during high-demand phases and capturing regenerative energy, thereby enhancing the hybrid system’s energy management capability and reducing battery fatigue.

The variation in instantaneous energy consumption for both BEV and HBEV configurations over the IM240 drive cycle is shown in Fig. 27. Energy spikes are observed during acceleration and regenerative braking phases due to transient power flow. By the end of the cycle, the total battery energy consumption is 51.3 Wh in the BEV, compared to 44.96 Wh in the HBEV, reflecting a 6.34 Wh reduction, or 12.36%, achieved through SC support. This notable reduction underscores the SC’s role in optimizing energy flow during dynamic events, ultimately enhancing the system’s overall efficiency and reducing battery usage.

The instantaneous speed response of the PMSM under the IM240 cycle, as illustrated in Fig. 28, shows that both BEV and HBEV configurations accurately track the reference speed despite frequent acceleration and deceleration events. The motor achieves a peak speed of 2534 RPM at 200 s, confirming the responsiveness and stability of the control system across dynamic operating conditions. No significant overshoot or lag is observed, indicating effective speed regulation in both configurations.

The instantaneous RMS stator voltage of the PMSM for both BEV and HBEV, as shown in Fig. 29, dynamically follows the motor speed profile. The inverter actively modulates the stator voltage in response to the reference speed to maintain torque demand and efficiency. A notable voltage spike of 183.2 V is observed at 185 s during a rapid transient, while the highest regular operating voltage of 174.84 V coincides with the peak motor speed of 2534 RPM at 200 s. These voltage fluctuations reflect the system’s response to varying load demands and confirm the robustness of voltage regulation across dynamic drive conditions.

The instantaneous Stator current of the PMSM for both BEV and HBEV configurations is presented in Fig. 30. Throughout the IM240 drive cycle, both systems exhibit identical current profiles, indicating that the supercapacitor integration primarily affects battery-side dynamics rather than motor-side operation. During peak acceleration at 160 s, the stator current reaches 79.6 A, while the highest decelerative current of 124.6 A is observed at 226 s. These peak values correspond to periods of intense energy exchange, highlighting the PMSM’s response to rapid torque demands during acceleration and effective regenerative braking during deceleration.

The instantaneous real power consumption of PMSM for both BEV and HBEV is shown in Fig. 31. Positive power values are observed during acceleration phases, indicating active power delivery to the motor, while negative values during deceleration reflect regenerative energy flow back to the sources. At 160 s, the peak motoring power reaches 17.06 kW, whereas the highest regenerative power of −20.76 kW occurs at 225 s. Notably, during the stationary interval between 92 s and 97.5 s—when the speed is zero—the motor consumes no power, confirming the proper functioning of the FOC strategy and demonstrating efficient dynamic response to varying drive conditions.

The instantaneous electromagnetic torque of the PMSM under IM240 conditions is presented in Fig. 32. Positive torque values are observed during acceleration phases, driven by the q-axis current component to meet dynamic load demands. In contrast, during deceleration, the q-axis current reverses, producing negative torque that enables regenerative braking by opposing the rotor motion. The peak motoring torque reaches 91.1 Nm at 160 s, while the highest regenerative torque of −139.4 Nm is recorded at 226 s, highlighting the PMSM’s responsive torque control across varying operating conditions. The consolidated performance metrics of the BEV and HBEV configurations under both EUDC and IM240 drive cycles are summarized in Table 5. Key parameters such as peak battery current, RMS current, voltage ripple, temperature, power, and energy consumption are compared, highlighting the improvements achieved through SC assistance.

**Fig. 23:**
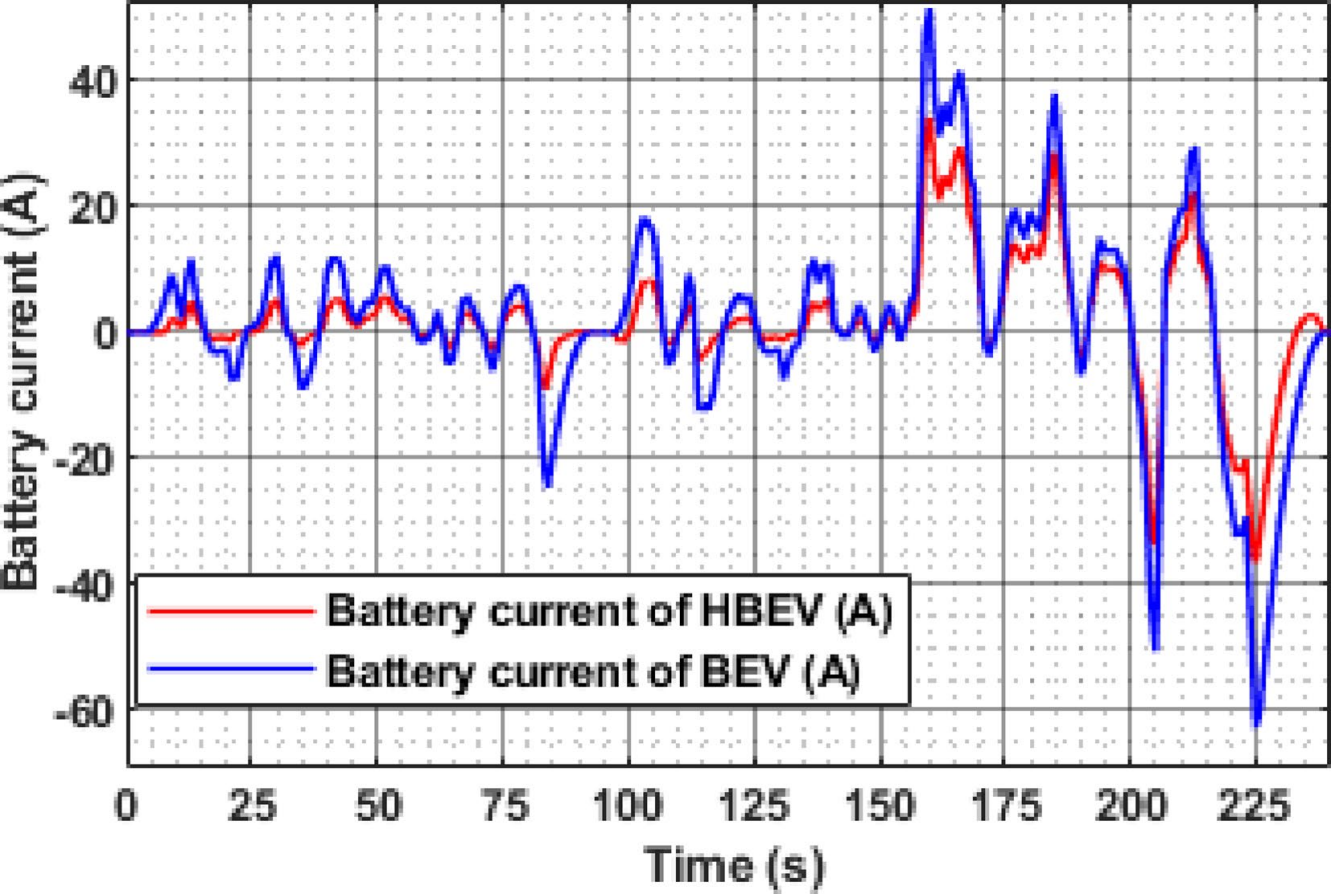
Battery current of BEV and HBEV.

**Fig. 24:**
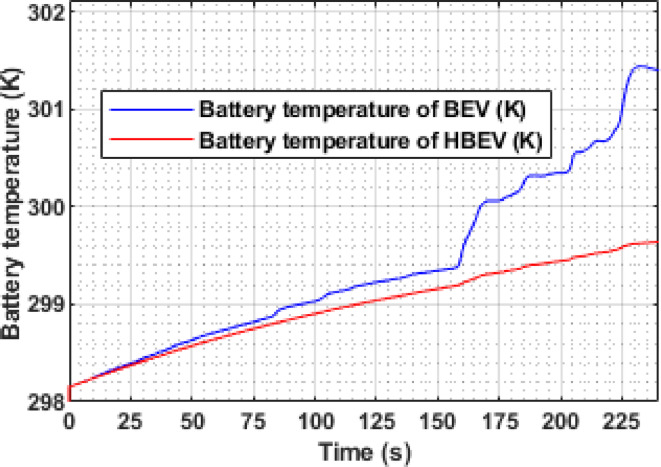
Battery temperature of BEV and HBEV.

**Fig. 25:**
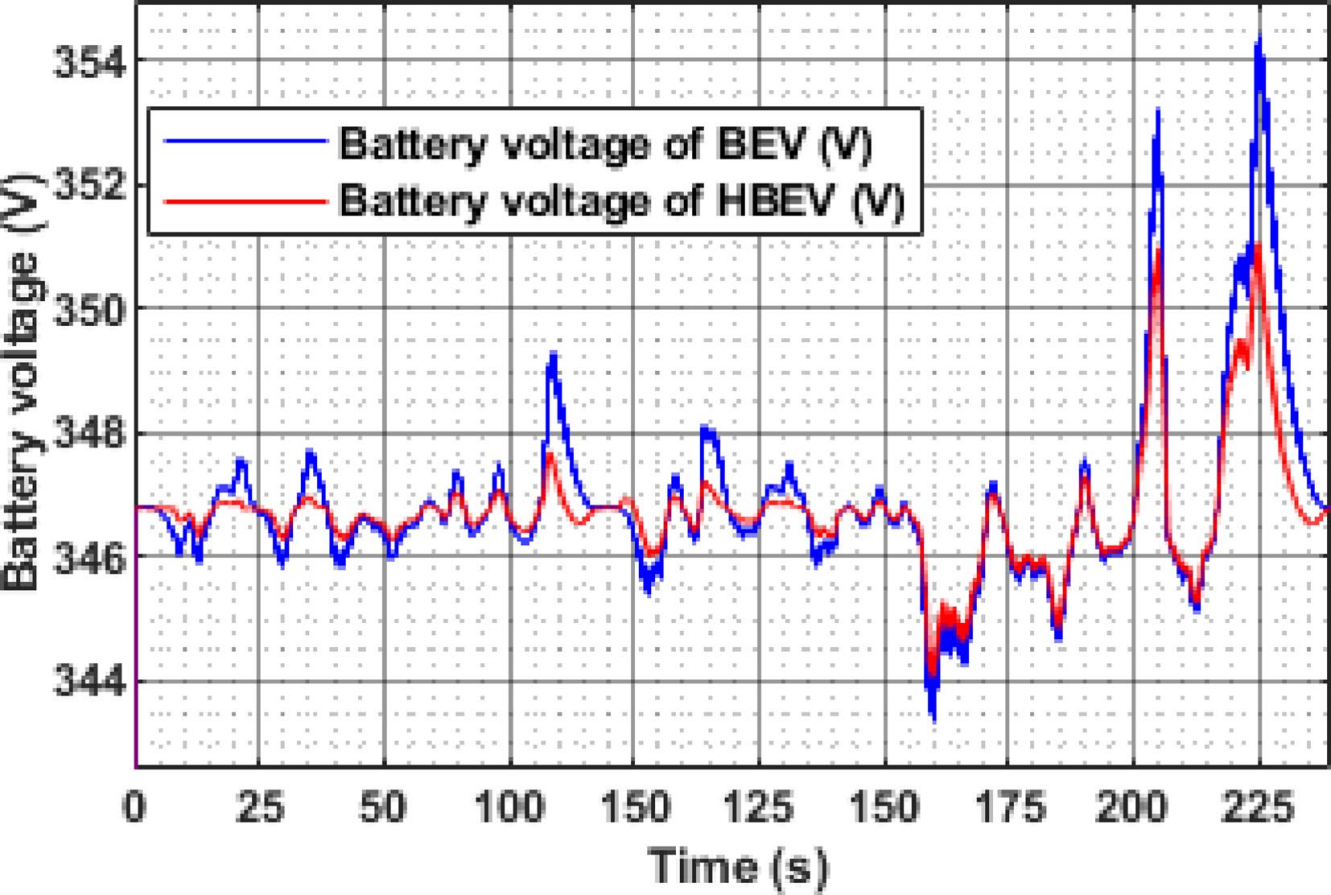
Battery voltage of BEV and HBEV.

**Table 5:** Performance comparison under EUDC and IM240.

Metric	EUDC			IM240		
	BEV	HBEV	Reduction (%)	BEV	HBEV	Reduction (%)
Peak battery current (A)	82.7	65.1	21.3	51.59	34.3	33.51
RMS battery current (A)	19.82	19.24	2.93	9.44	8.83	6.38
Battery voltage ripple (%)	3.87	2.28	1.59	3.17	1.99	1.18
Peak battery temperature (K)	302.2	300.8	0.463	301.4	299.64	0.584
Peak battery power (kW)	27.92	22.49	18.1	17.1	11.7	31.6
Energy consumption (Wh)	547.5	516	5.75	51.3	44.96	12.36

**Fig. 26:**
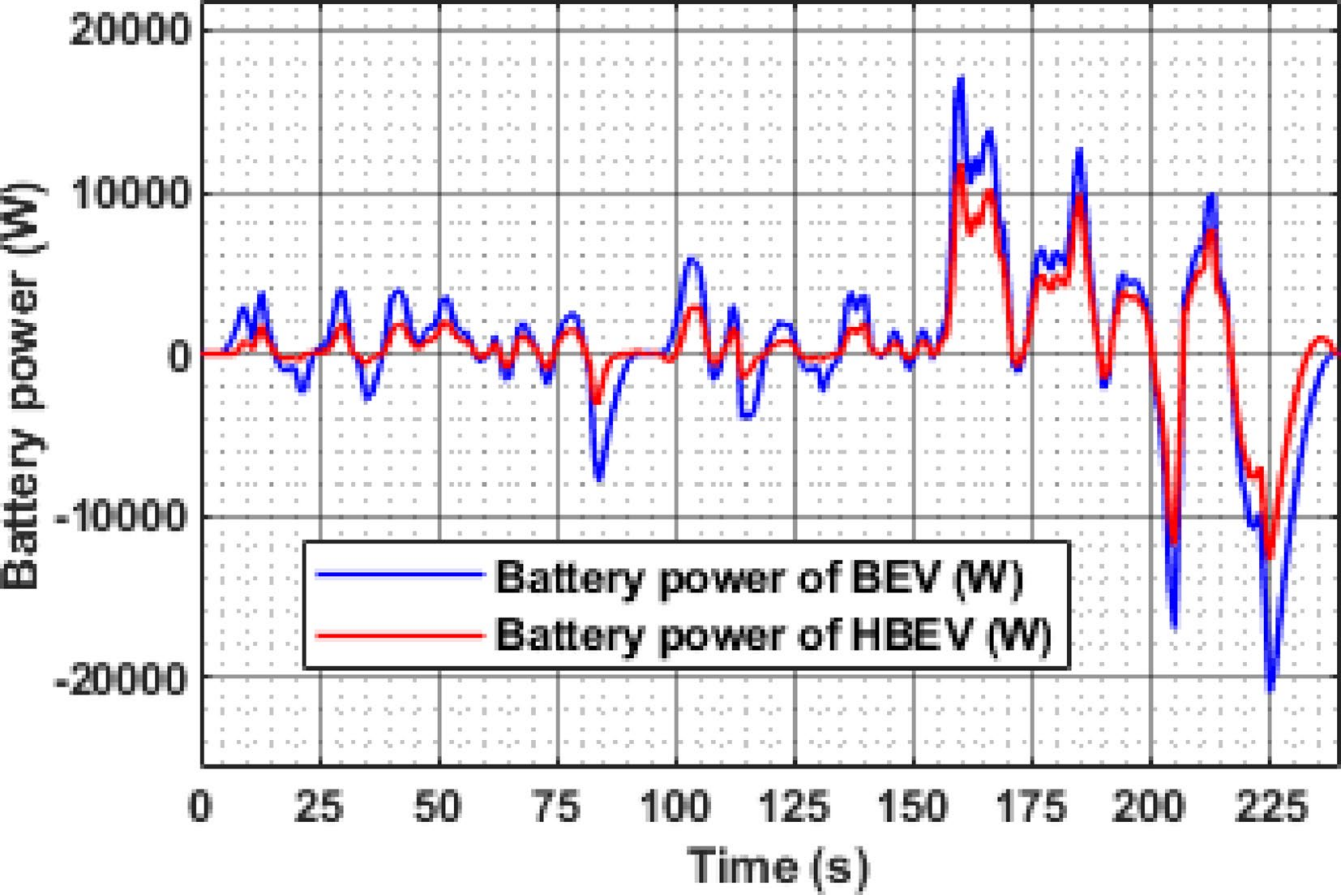
Battery power of BEV and HBEV.

**Fig. 27:**
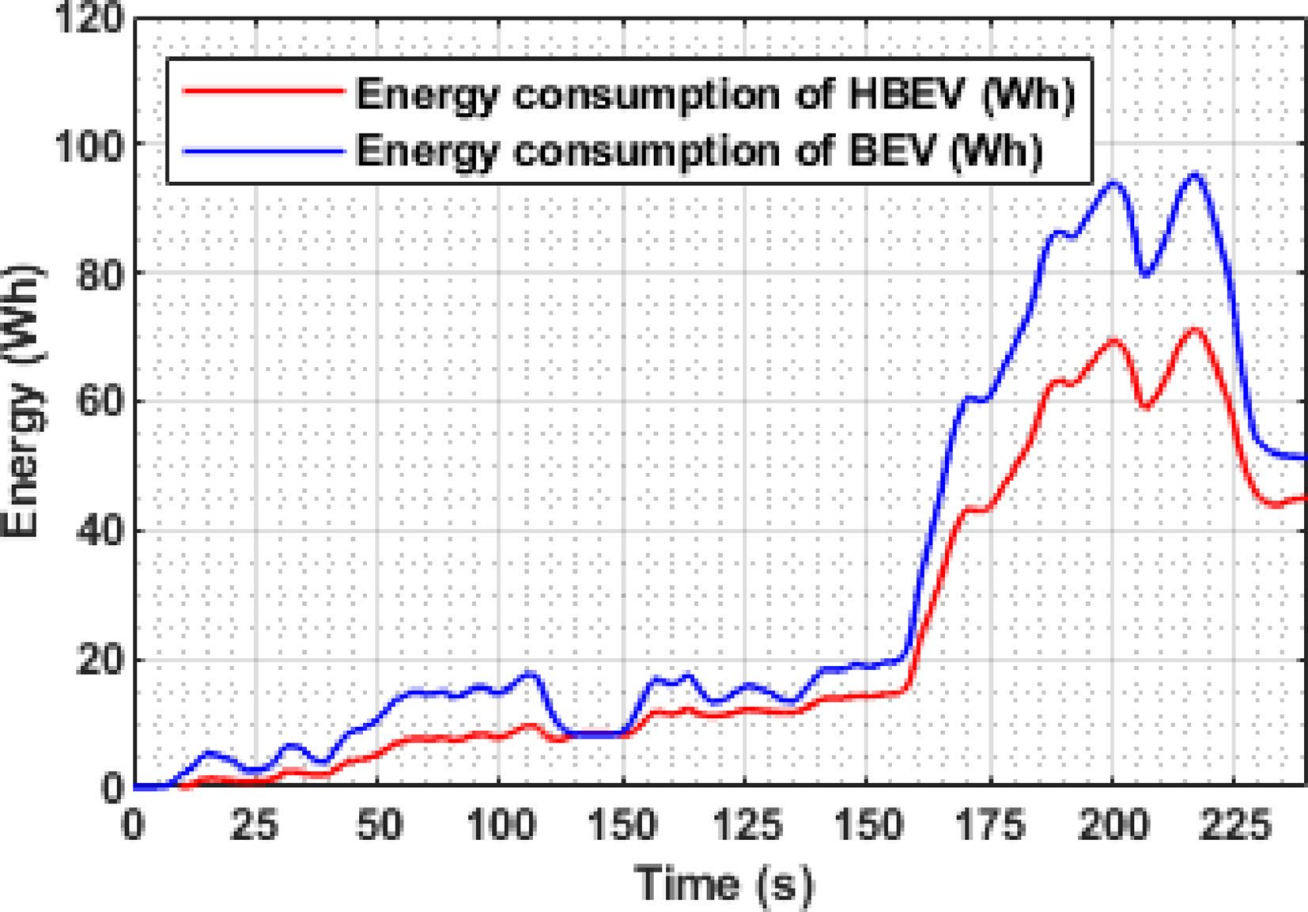
Battery energy consumption of BEV and HBEV.

**Fig. 28:**
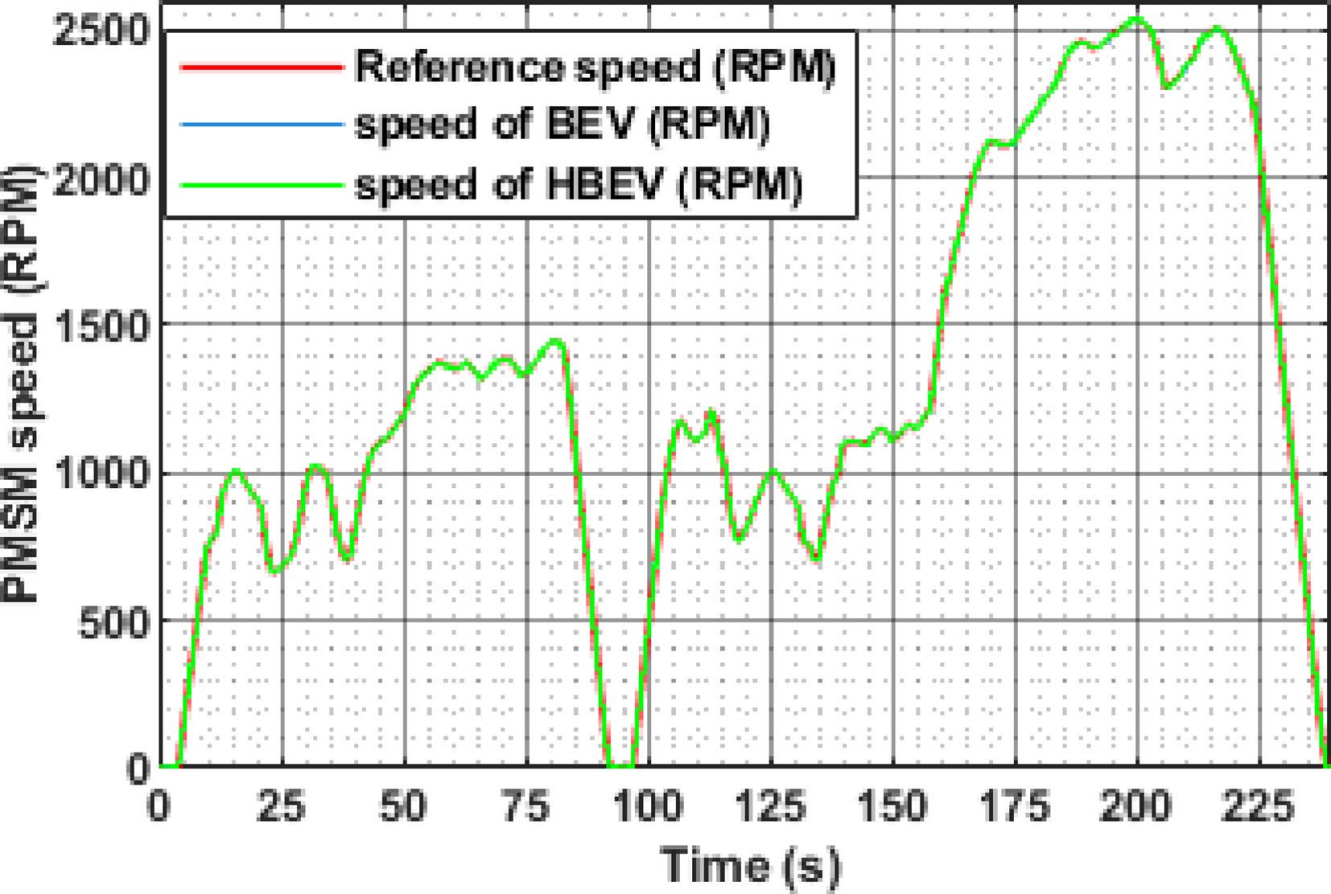
Reference and actual speed of PMSM.

**Fig. 29:**
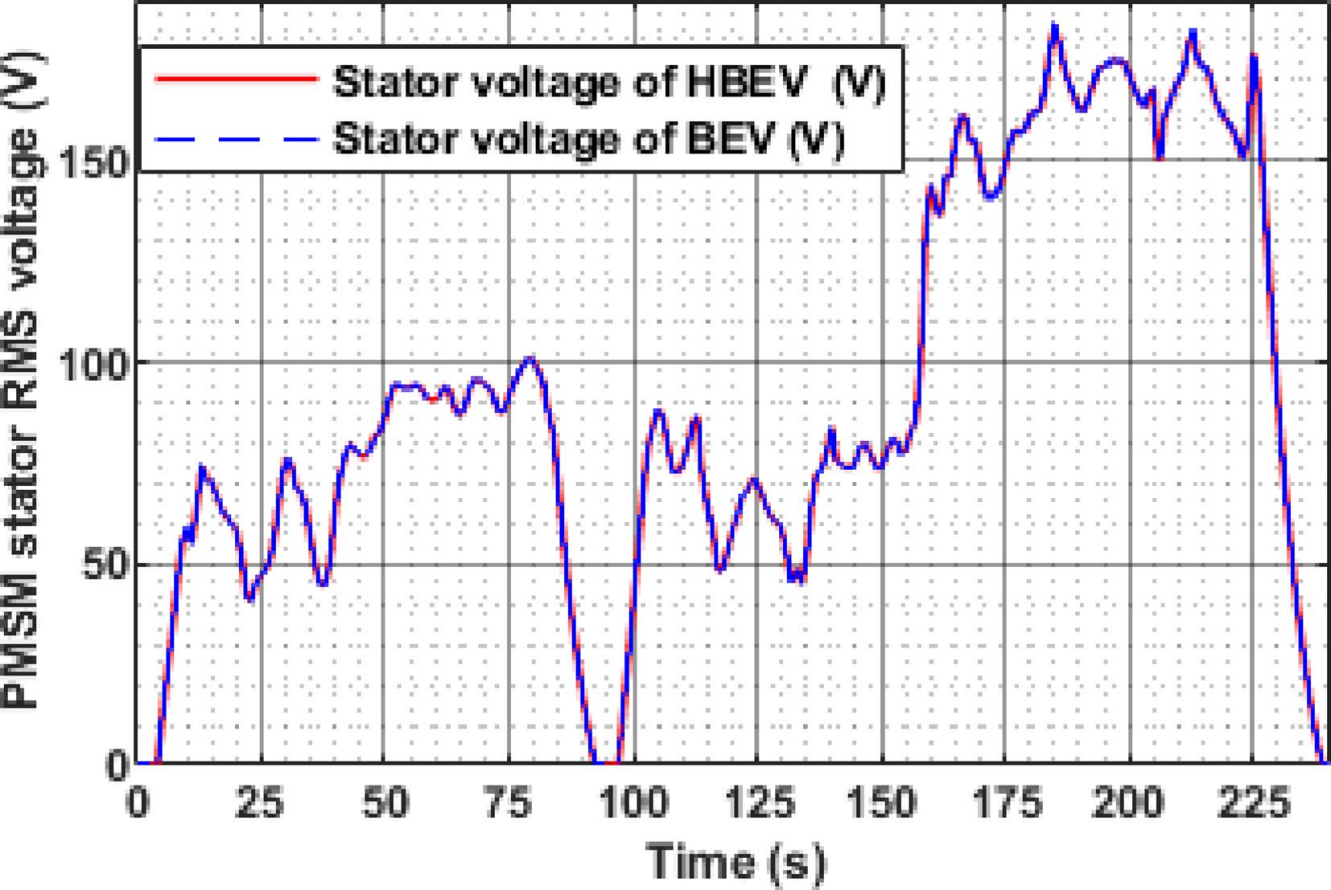
Stator voltage of PMSM.

**Fig. 30:**
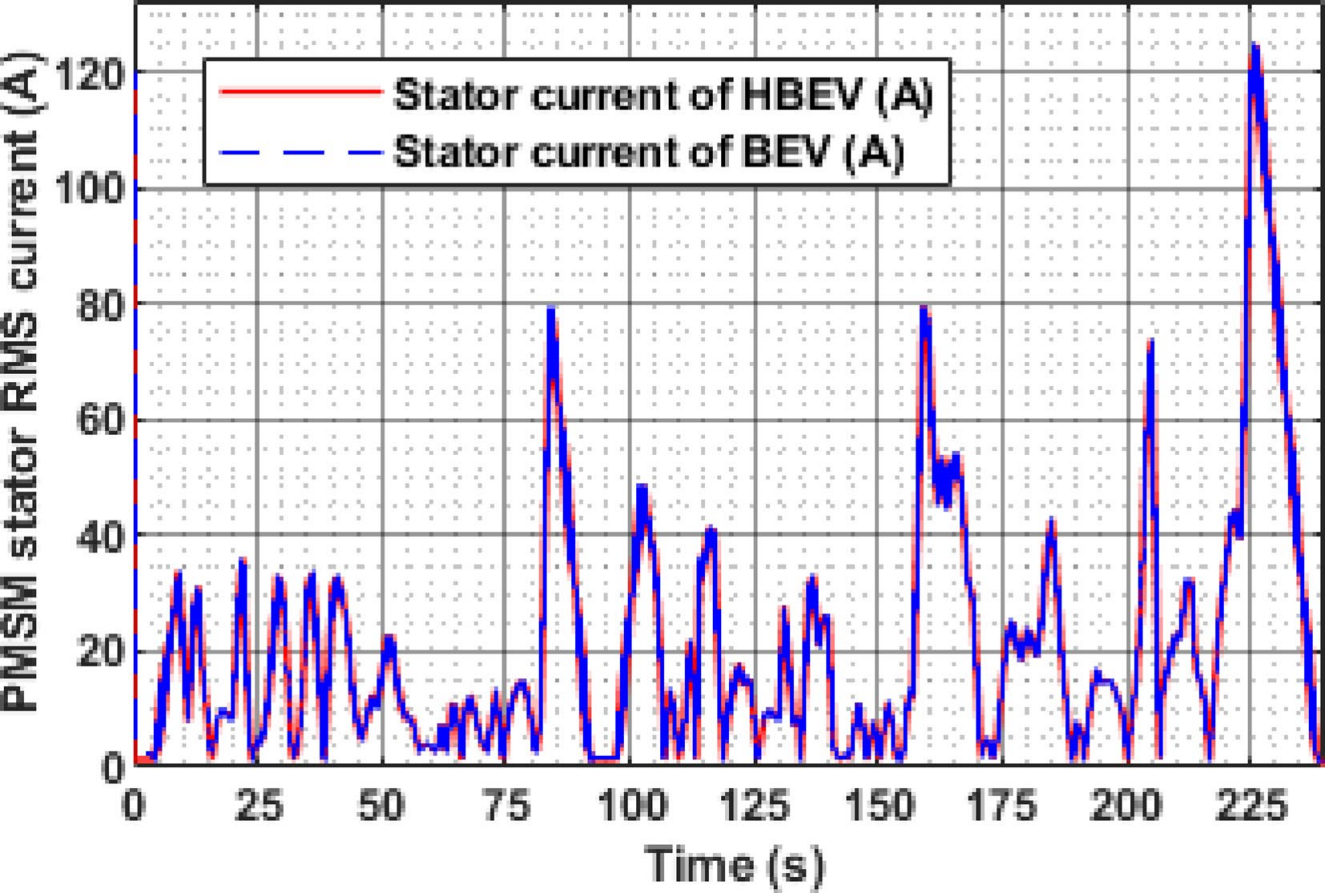
Stator current of PMSM.

**Fig. 31:**
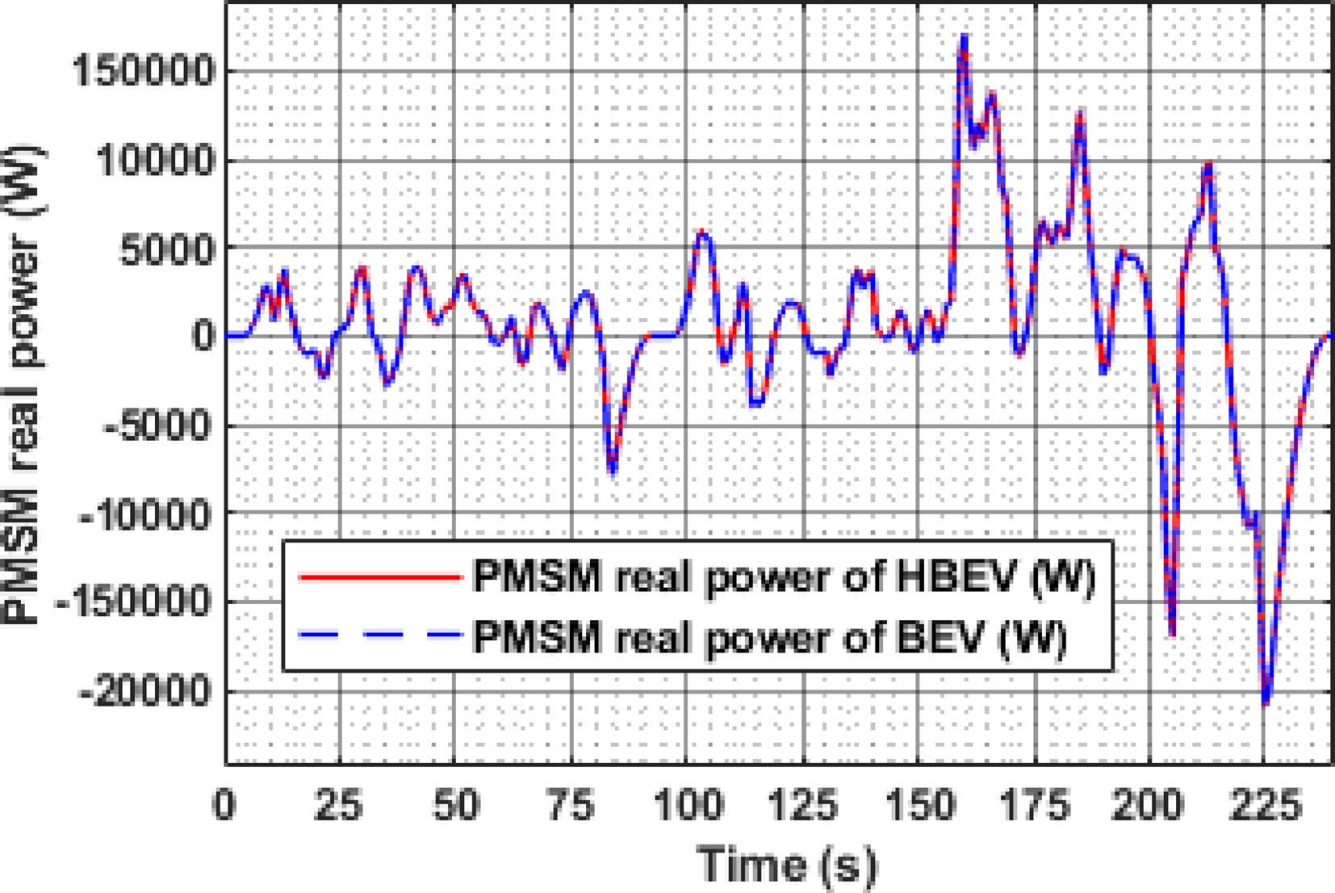
Real power of PMSM..

**Fig. 32:**
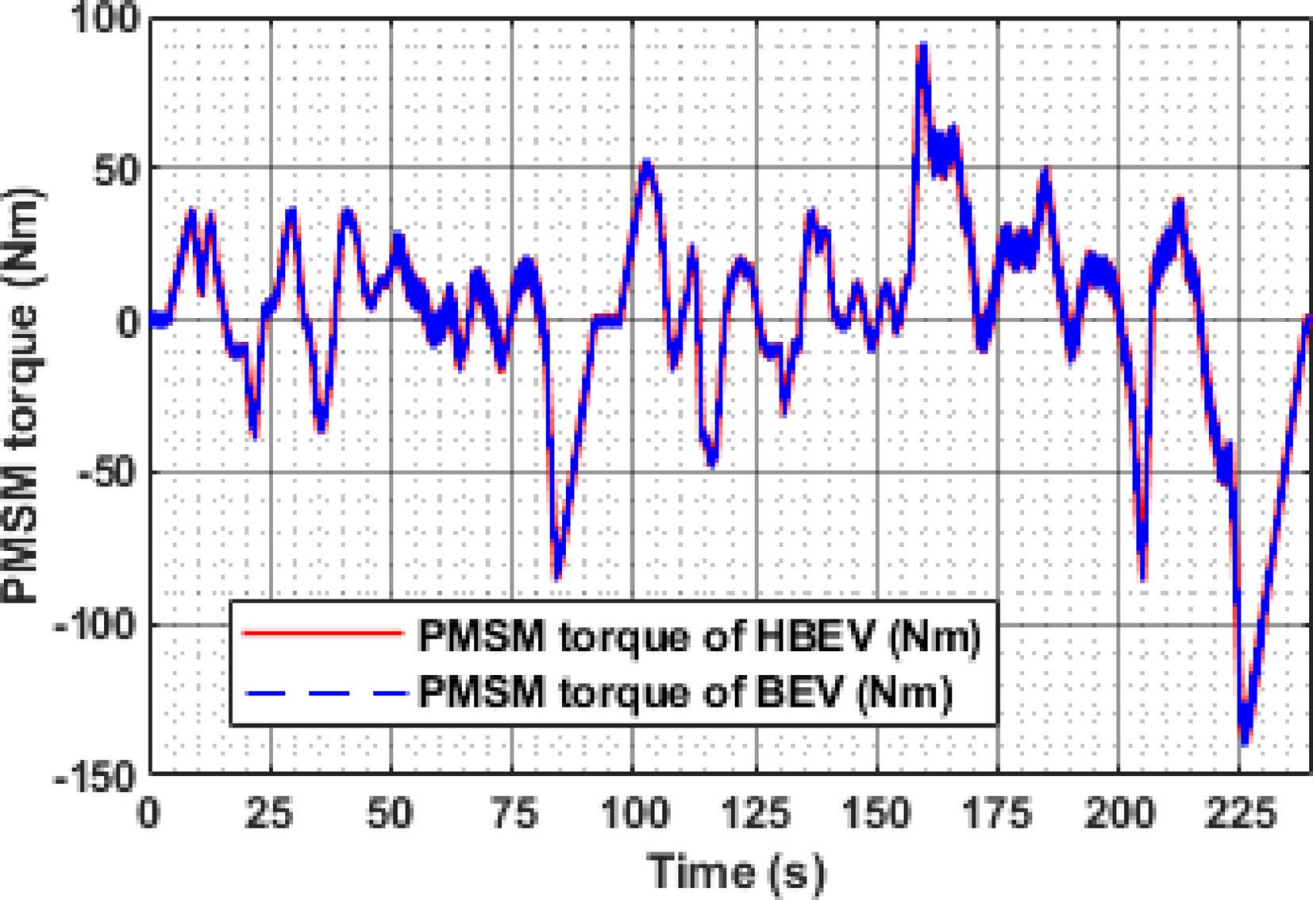
Electromagnetic torque of PMSM.

Table 6 provides a comparative overview of RMS and peak current reduction achieved by various energy management strategies across different drive cycles. Techniques such as ANFIS + DPR and GWO + SVM show moderate RMS current reductions ranging from 3.3 to 11.43%, while WT and fuzzy logic-based methods report significant peak current reductions of over 50% under highly transient cycles like NYCC. Similarly, AFNN-based strategies have achieved peak current mitigation up to 51% in UDDS.

In comparison, the proposed LSTM + ONNX framework demonstrates a balanced improvement in both RMS and peak current performance. Under the IM240 cycle, it achieves a 6.38% reduction in RMS current and 33.51% reduction in peak current, while under the more aggressive EUDC profile, it delivers 2.92% RMS and 21.3% peak current reduction. Although slightly lower in magnitude than some heuristic approaches under specific conditions, the proposed method offers consistent, real-time control adaptability with less reliance on drive cycle-specific tuning—highlighting its potential for generalizable implementation in embedded battery-supercapacitor hybrid systems.

**Table 6:** Comparative analysis of RMS and peak current reduction performance across various EMS methods.

Method/Approach	Drive cycle	RMS current reduction (%)	Peak current reduction (%)
ANFIS + DPR[36]	FTP	11.43	-
	Artemis	5	-
	WLTP class	3.3	-
WT + FLC[61]	NYCC	-	58.2
WT [61]	NYCC	-	50.1
GWO + SVM[44]	Manhattan	8.8	-
AFNN[6]	UDDS	-	51
	Random	-	45.6
LSTM + ONNX (Proposed)	IM240	6.38	33.51
	EUDC	2.92	21.3

## Conclusion

This work proposes a novel ONNX-deployed LSTM-based strategy for real-time SC current prediction in BEV and HBEV. By enabling adaptive energy distribution between the battery and SC, the method improves powertrain efficiency and reduces battery stress under dynamic driving conditions.

Simulation results using a MATLAB Simscape model of the Nissan Sakura EV demonstrate significant improvements. Under the EUDC cycle, SC assistance reduced battery peak current by 21.3%, peak power by 18.1%, and energy consumption by 5.75%. For the IM240 cycle, peak current dropped by 33.5%, peak power by 31.6%, and energy usage by 12.36%. These outcomes validate the effectiveness of the proposed LSTM-based SC current control framework in optimizing battery utilization and enhancing overall system performance.

However, the model is trained only on standard drive cycle data, which may limit its generalization to highly variable urban conditions. The fixed SC configuration does not account for component aging or adaptive control. Furthermore, the absence of an optimization layer restricts the ability to jointly minimize battery degradation, energy loss, and power ripple.

Future work will focus on expanding the input features such as battery SoC, SC voltage, integrating adaptive SC sizing strategies, and embedding real-time optimization to support intelligent and robust energy management across diverse drive scenarios.

## Data Availability

Data availability: The datasets used and/or analyzed during the current study available from the corresponding author on reasonable request. The confidentiality of this research necessitates the restriction of some raw data. However, the minimal dataset necessary to replicate the findings presented in this manuscript is provided.
